# A Randomised, Double Blind, Placebo-Controlled Pilot Study of Oral Artesunate Therapy for Colorectal Cancer^☆^^☆☆^

**DOI:** 10.1016/j.ebiom.2014.11.010

**Published:** 2014-11-15

**Authors:** Sanjeev Krishna, Senthil Ganapathi, Irina Chis Ster, Mohamed E.M. Saeed, Matt Cowan, Caroline Finlayson, Hajnalka Kovacsevics, Herwig Jansen, Peter G. Kremsner, Thomas Efferth, Devinder Kumar

**Affiliations:** a Institute of Infection and Immunity, Department of Pathology, United Kingdom; b Department of Surgery, St. George's, University of London, Cranmer Terrace, SW17 0RE, United Kingdom; c Department of Pharmaceutical Biology, Johannes Gutenberg-University, Staudinger Weg 5, 55128 Mainz, Germany; d Department of Gastroenterology, Surrey and Sussex Healthcare NHS Trust, East Surrey Hospital, Canada Avenue, Redhill, Sussex RH1 5RH, United Kingdom; e Dafra Pharma nv, 2300 Turnhout, Belgium; f Institut für Tropenmedizin, Universitätsklinikum Tübingen, Wilhelmstraße 27, D-72074 Tübingen, Germany

**Keywords:** Colorectal cancer, Artesunate, Dihydroartemisinin, Ki67, Neutropaenia

## Abstract

**Background:**

Artesunate is an antimalarial agent with broad anti-cancer activity in *in vitro* and animal experiments and case reports. Artesunate has not been studied in rigorous clinical trials for anticancer effects.

**Aim:**

To determine the anticancer effect and tolerability of oral artesunate in colorectal cancer (CRC).

**Methods:**

This was a single centre, randomised, double-blind, placebo-controlled trial. Patients planned for curative resection of biopsy confirmed single primary site CRC were randomised (n = 23) by computer-generated code supplied in opaque envelopes to receive preoperatively either 14 daily doses of oral artesunate (200 mg; n = 12) or placebo (n = 11). The primary outcome measure was the proportion of tumour cells undergoing apoptosis (significant if > 7% showed Tunel staining). Secondary immunohistochemical outcomes assessed these tumour markers: VEGF, EGFR, c-MYC, CD31, Ki67 and p53, and clinical responses.

**Findings:**

20 patients (artesunate = 9, placebo = 11) completed the trial per protocol. Randomization groups were comparable clinically and for tumour characteristics. Apoptosis in > 7% of cells was seen in 67% and 55% of patients in artesunate and placebo groups, respectively. Using Bayesian analysis, the probabilities of an artesunate treatment effect reducing Ki67 and increasing CD31 expression were 0.89 and 0.79, respectively. During a median follow up of 42 months 1 patient in the artesunate and 6 patients in the placebo group developed recurrent CRC.

**Interpretation:**

Artesunate has anti-proliferative properties in CRC and is generally well tolerated.

## Highlights

• Artesunate is a cheap and orally available, widely used antimalarial drug that has anticancer properties in *in vitro* models
• Tested the effects of artesunate on colorectal cancer in patients before they have had surgery to remove the tumour
• Results from this pilot study are promising and support further studies to explore anticancer effects of artemisinins

## 1 Introduction

Colorectal cancer (CRC) contributes 9–10% of the annual global cancer burden in men (746,000 cases) and women (614,000 cases) (Ferlay et al., 2012). In the UK, 110 new cases are diagnosed daily, with older patients particularly at risk of death (UK CR, 2014) and with > 50% of newly diagnosed cases having locally advanced disease (T3/T4). Resection is the only curative treatment for non-metastatic CRC but this has to be combined with neo-adjuvant chemo- and/or radio-therapy, to downstage more advanced presentations.

Prognosis with best available treatments does not increase disease free or overall survival beyond ~ 60% at 5 years after diagnosis. For most patients, access to advanced treatment modalities is lacking, too expensive to be widely available, or associated with significant morbidity thereby further compromising their survival. There is therefore a continuing and urgent need to develop new, cheap, orally effective and safe CRC therapies. One approach is to study existing drugs that already have some anticancer properties in experimental settings, and to assess their safety and efficacy in *in vivo* studies.

Artesunate is derived from artemisinin, which is extracted from *Artemisia annua* L. and is a widely used antimalarial that can be administered by oral, rectal and parenteral routes (Gomes et al., 2009, Kremsner and Krishna, 2004, Kremsner et al., 2012, Nealon et al., 2002, Hien et al., 1992, Hien et al., 1994, Jiang et al., 1982). Soon after the isolation of artemisinin by a Chinese government's programme, the anticancer properties of artemisinins were first reported (Efferth et al., 2007, Krishna et al., 2008). Subsequently, many studies of artemisinins using *in vitro* and animal models have confirmed their remarkable capacity to exert broad anti-cancer effects (Efferth et al., 2007). They reduce cell proliferation and angiogenesis and trigger apoptosis (Anfosso et al., 2006, Efferth et al., 1996, Efferth et al., 2001).

There have only been isolated case reports in humans of anti-cancer effects of artemisinins (reviewed Krishna et al., 2008). These include cases of metastatic uveal melanoma (Berger et al., 2005) laryngeal squamous cell carcinoma (Singh and Verma, 2002) and pituitary macroadenoma (Singh and Panwar, 2006). An open-label Chinese study treated non-small cell lung cancer patients and showed prolonged time to cancer progression compared with controls when artesunate was added to conventional treatment (Zhang et al., 2008), but no benefit on mortality. An open-label pilot study of patients receiving artesunate for advanced cervical cancer suggested that it was well tolerated and improved symptoms (Jansen et al., 2011). There has been a phase II trial on the activity of artesunate in non-resectable tumours of dogs (Rutteman et al., 2013) and efficacy of extracts of *A. annua* in 5 veterinary sarcomas (Breuer and Efferth, 2014). This study examines anti-CRC effects and tolerability of artesunate used as monotherapy in a rigorous study design.

## 2 Methods

### 2.1 Ethics

The trial was approved by Wandsworth Ethics Committee (Wandsworth UK, Ref: 08/H0803/3) and was registered (ISCRTN05203252).

### 2.2 Trial Design

This was a single-centre, double-blind, placebo-controlled trial with balanced randomisation of patients (1:1) conducted at the St George's University of London, UK and St. George's Healthcare NHS Trust.

### 2.3 Participants for Inclusion

Eligible participants were with biopsy confirmed single primary site colorectal adenocarcinoma; aged 21–90 years; with all stages amenable to surgical treatment and not requiring neoadjuvant treatment; with planned curative resection; and with written, informed consent.

### 2.4 Exclusion Criteria

These were: contraindication to use of artesunate due to hypersensitivity; pregnancy; history of hearing or balance problems; immunosuppression or concomitant medication known to interact with artesunate (see below); weight < 50 kg or > 100 kg; severe anaemia (haemoglobin < 8 g/dL); other planned intervention, apart from standard of care; inability to give informed consent; inability or unwillingness to take effective contraception in women of child-bearing age; chronic kidney disease of NKF D/QOFI stage 3 or above (eGFR < 60 mL/min); bilirubin > 2 of the upper limit of normal without haemolysis or known chronic liver disease.

### 2.5 Recruitment

Recruitment was at St George's Healthcare NHS Trust in London from 9 March 2009 to 15 October 2012.

### 2.6 Interventions

Patients received two weeks of experimental medication (artesunate or placebo) just before surgery and standard care. Artesunate (Arinate® 100 mg) was manufactured by Famar Italia S.p.A and matching placebo tablets were manufactured by MPF in The Netherlands under a manufacturing licence in accordance with EU cGMP certified by Dafra Pharma (Belgium). Study medication was packaged, labelled and certified by B&C CliniPack (Belgium) and was in pack sizes of 30 × 100 mg and was received, stored and dispensed by the Pharmacy at St George's Healthcare NHS Trust.

The dose of artesunate for the study was 200 mg orally, daily for fourteen days, with medication stopped 48–72 h prior to surgery.

Medication was provided in blister packs with one patient box provided 14 doses, sufficient for the duration of the study.

There was no delay in surgery if patients entered into this study, nor any other change in clinical management, and the 62 day rule (requiring treatment within this time period after confirmation of diagnosis) was strictly adhered to.

### 2.7 Outcomes

The primary endpoint of the trial was the presence or absence of significant apoptosis in the epithelial cells of the tumour specimen defined as > 7% of cells with apoptotic features.

Secondary outcomes included seven immunohistochemical stains applied to the paraffin-embedded tumour specimens and quantified in both epithelial cells and fibroblasts: vascular endothelial growth factor (VEGF), c-MYC status and EGF-receptor status; microvessel density determining the quantity of the cluster of differentiation 31 (CD31) protein; proliferative activity assessed with Ki67 staining and p53 tumour suppressor protein expression. Each stain in each patient was generally evaluated in 6 microscopic areas with a semiautomatic system (in a few cases 7 or 8 areas were evaluated, and in some – especially for fibroblasts – measurements less than 6 or no areas could be evaluated).

### 2.8 Blood Samples

Three blood samples were taken: (1) at baseline, (2) after one week of medication (following protocol amendment for enhanced safety monitoring) and (3) after ending the two week medication (just before surgery). In each sample the safety measures included assay of potassium, sodium, creatinine, urea, albumin, alkaline phosphatase, ALT, bilirubin, haemoglobin, platelet count and white cell count. Carcinoembryonic antigen (CEA), was monitored where available in patients at baseline and after randomization.

### 2.9 Secondary Outcomes

These were measures of safety and tolerability (both clinical and laboratory) according to conventional criteria assessed by comparing baseline blood test results and those during or after treatment and anticancer efficacy (with markers described above).

#### 2.9.1 Changes to Outcomes

There were no changes to predefined endpoints.

### 2.10 Sample Size

An indicative sample size calculation, given the pioneering nature of this pilot study, was carried out on the primary outcome before starting the trial based on the assumption that colorectal cancer is unlikely to exhibit significant apoptosis if untreated. Most patients in the placebo group (more than 95%) were anticipated to have less than 7% of cells with apoptotic features. The majority of patients (greater than 60%) in the artesunate group were anticipated to have significant apoptosis. This large difference was derived from published baseline estimates of apoptotic indices (Yamamoto et al., 1998, Ikenaga et al., 1996, Bendardaf et al., 2003). With equal group sizes a sample size of 2*11 was estimated to have 80% power and accepting a Type I error of 5% for superiority, bearing in mind that in most pilot studies the aim is to demonstrate proof-of-concept (Arain et al., 2010) rather than exclusively test a hypothesis.

### 2.11 Randomisation

Subjects were randomised to receive either artesunate or placebo in equal numbers. Randomisation was performed using a computer-generated code, and results supplied in opaque and sealed envelopes by Dafra Pharma. After enrollment and allocation of the next study number in the series, participants were given their randomization pack by a pharmacist. Copies of the key to the randomisation codes were held by the Clinical Trials Pharmacist only and allocation and concealment steps were performed in Belgium. The code was not opened to investigators, patients, data collectors until the data collection ended, histological results had been analysed and datasets were locked.

### 2.12 Sequence Generation and Allocation Concealment Mechanism

Study medications were pre-packed in blister packs and consecutively numbered for each participant according to the randomisation schedule. Each participant was assigned an order number once they had consented to the study and after eligibility checks, and they received the medication pack with the corresponding randomization number.

### 2.13 Blinding

It became necessary to unblind the allocation to 2 participants during the course of the study at the request of the MHRA after receipt of notification of Adverse Events. Blinding was maintained for all investigators and codes were supplied by the Sponsor's office (SGUL) to the MHRA.

### 2.14 Data Collection and Structure

**Fig. 1:**
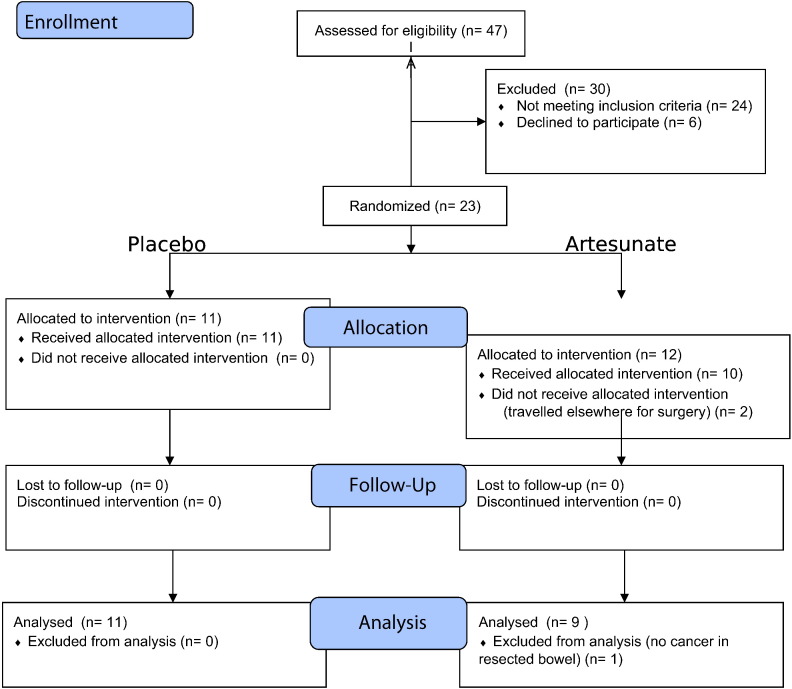
Patient flow diagram. Assessment for eligibility was recorded after 6 patients had been randomised, so that these added to the 17 patients randomised after screening give the total number of randomised patients (n = 23).

Immunohistopathological data generated multiple measurements per individual as the number of slides differed according to the sizes of the tumours. Each measurement represents an estimate of staining of cancer cells found on a slide with 0 denoting no staining observed on a section. Therefore, the dataset inherits a hierarchical structure with patients at level one and within individual measurements as level two. With the exception of the three individuals (Fig. 1) there are no records considered missing for statistical analysis.

### 2.15 Exploratory Statistical Analysis

The nature of all data variables have been graphically assessed and summarised accordingly with means/medians standard deviation for continuous data or proportions for binary data. Correlations were explored with Spearman's coefficient.

### 2.16 Immunochemistry Results and Inferential Data Analysis

A random effects (variance components) model was employed for immunochemistry data to capture their variabilities correctly, given their inherent hierarchical structures.

Patients were followed-up with CEA measurements every 6 months and annual CT scans for disease recurrence. Time since surgery to the first disease recurrence has been modelled with survival analysis. Patient CRC06 has been included in survival analysis and although there were no samples obtained for immunochemistry, the patient was known to have survived. Patient CRC13 was initially randomised to artesunate but was included in the survival analysis as placebo as s/he did not receive any drug (as per protocol analysis). Patient CRC21 was deemed as missing and sensitivity analysis including them in either artesunate or placebo groups is provided. The Cox proportional hazard's (PH) model has been applied to investigate the hazard ratio of disease recurrence for artesunate compared with placebo. and their pointwise 95% confidence intervals are provided for each treatment group.

### 2.17 Statistical Inference

Model based Bayesian analysis and classical frequentist approaches have been applied as appropriate. Frequentist statistical significance is conventionally associated with p-values less than 0.05 with uncertainty of parameters assessed by the 95% confidence intervals (CI). Parameter estimates in Bayesian inference are summarised by their posterior means and the corresponding 95% credible intervals (CrI). Initially, no prior knowledge was assumed for the parameter that quantifies the treatment effect, *i.e.* the difference between the group means in terms of immunochemistry measurements and the inference that has been drawn. If prior information exists, then it is appropriate to assess how the parameter values change based on this evidence. Ki67 and CD31 were the only stains with prior anti-CRC information available (Li et al., 2007, Jansen et al., 2011). A sensitivity analysis was conducted with Ki67. Statistical software used included OpenBUGS (Thomas et al., 2006) STATA (StataCorp. 2013. *Stata Statistical Software: Release 13*. College Station, TX: StataCorp LP) and R (Team RDC, 2011).

## 3 Results

### 3.1 Participant Flow

Fig. 1 summarises patient flows. 12 patients were randomised to receive artesunate and 11 to placebo. 2 patients did not receive artesunate despite randomization (one travelled for surgery outside the UK and could not be followed up, and the drug expiry date for the relevant batch had been reached when the other patient attended). One artesunate recipient could not be evaluated for the primary endpoint because no tumour was identified by histology after operation. Recruitment ended after the planned numbers were randomised.

### 3.2 Baseline Data

Baseline demographic and clinical characteristics are summarised in Table 1. There is comparability between groups, including for Dukes' staging.

### 3.3 Primary Outcome

55% of placebo recipients and 67% of artesunate treated patients achieved the primary outcome (patients in whom the proportion of apoptotic cells was > 7%). In designing this trial, it was assumed that only ≤ 1/11 patients receiving placebo would have > 7% of tumour cells displaying apoptosis. The unpredicted higher baseline values in placebo recipients precluded detection of an artesunate effect in the primary outcome. Staining results for Tunel as a continuous variable are in Table 2.

### 3.4 Secondary Outcomes

#### 3.4.1 Immunohistochemistry Analyses

Table 2 presents analysis of immunohistochemistry results. A random effects model scaled linearly provides the posterior distributions of the group means, their 95% CrI and their estimated differences between groups. The estimated posterior distributions of differences between treatment groups lie on both sides of 0 for most measurements suggesting that the two treatment groups do not differ markedly for most analysed markers.

**Fig. 2:**
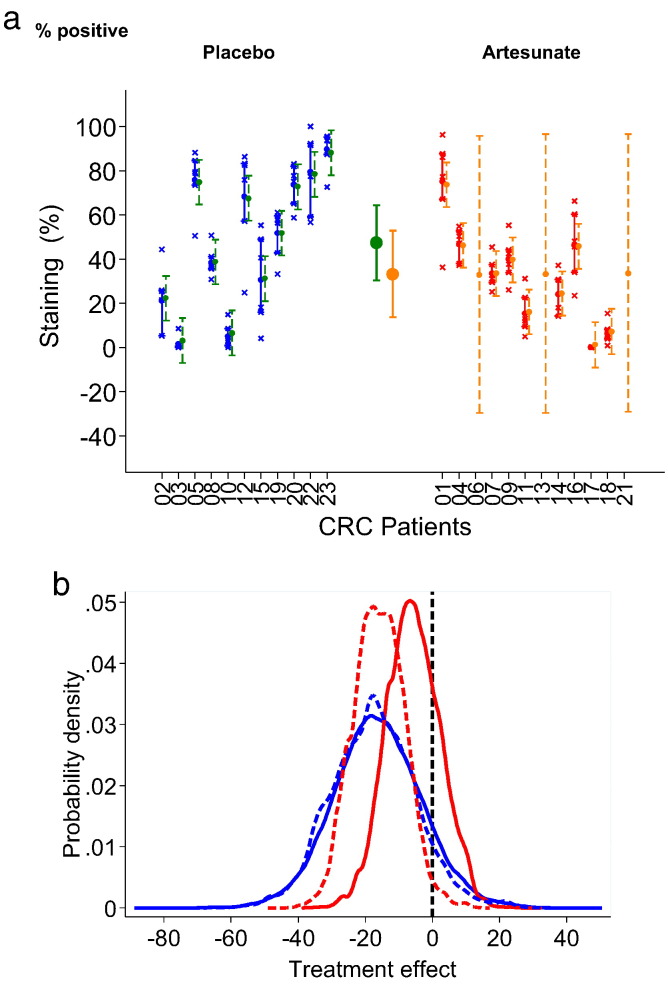
2a Ki67 staining in treatment groups. Individual results for Ki67 epithelial cell staining (% positive), the grand means in each treatment group as predicted by the random effects model and individual predications are displayed. Interquartile intervals are presented for the raw measurements and the 95% credible intervals correspond to the individual and group predicted means. This analysis has been carried out under *missing at random assumption* (MAR) with large uncertainty around the individual means of missing individuals, as expected and shown for 3 individuals randomised to receive artesunate but not able to be analysed for reasons given in results. These results correspond to non-informative prior assumption with regards to the difference between the two groups. 2b Sensitivity analysis to various prior information on the difference between the two groups with respect to Ki67 — the analysis has been justified by published experimental results. The probability that the difference between artesunate and placebo is negative remains high even under a skeptical prior of no effect (0.77).

Interestingly, the probability of a reduction in Ki67 staining after artesunate is 89–92% (Table 2; a result that is 1–0.11, because Ki67 is downregulated) using a non-informative prior on the Ki67 difference between groups and resulting in a posterior difference of − 16 (− 42, 10) for treatment. A similar result is confirmed if the confidence around the parameter value is increased as illustrated in Fig. 2a. With an optimistic informative prior for artesunate of − 15 (N (− 15,10)), the probability of an artesunate effect increases to 97% (1–0.03; Table 2), by altering the parameter distributions as summarised in Fig. 2b.

**Fig. 3:**
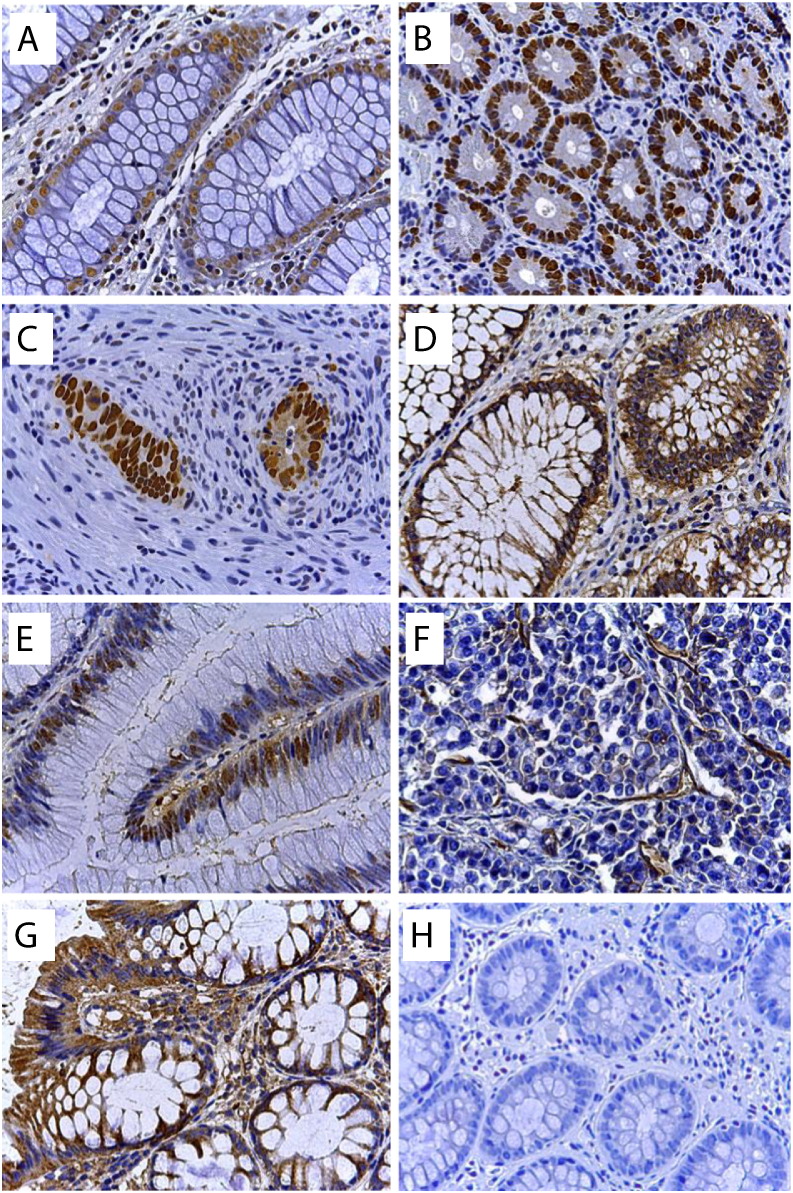
Immunohistochemical staining of biomarkers in colorectal cancer. (a) Detection of apoptotic cells by the Tunel assay, (b) Ki67, (c) p53, (d) EGFR, (e) c-MYC, (f) CD31, (g) VEGF, (h) negative control (without primary antibody). Magnification: × 250. For determination of protein expression the UltraVision polymer detection method (kit from Thermo Fisher Scientific GmbH, Dreieich, Germany) was used as detailed in Supplementary methods. The immunostained slides were scanned by Panoramic Desk (3D Histotech Pannoramic digital slide scanner, Budapest, Hungary) and interpreted (Quantification of immunostained slides) by panoramic viewer software (NuclearQuant and membraneQuant, 3DHISTECH) in which positive stained nucleus or membrane were counted in each defined annotated area. Evaluation parameters included number of overall detected objects (nucleus or membrane) in each annotated area, average of positivity and intensity. Nuclear stainings (Ki67, p53, c-MYC, TUNEL) were quantified using the Nuclear Qant software and Membrane-bound and cytosolic stainings were quantified by the MembraneQuant software (3D histoQuant). Results are in Table 2.

To complete this analysis, we included a skeptical informative prior for this parameter (N (0, 10)). Despite this, the probability of an artesunate effect remains at a probability of 0.77 with a true mean value estimated at − 16 (− 22,10; Table 2). CD31 also provided a high probability (0.79) for a treatment effect. Representative immunostainings are shown in Fig. 3.

#### 3.4.2 Survival Analysis

**Fig. 4:**
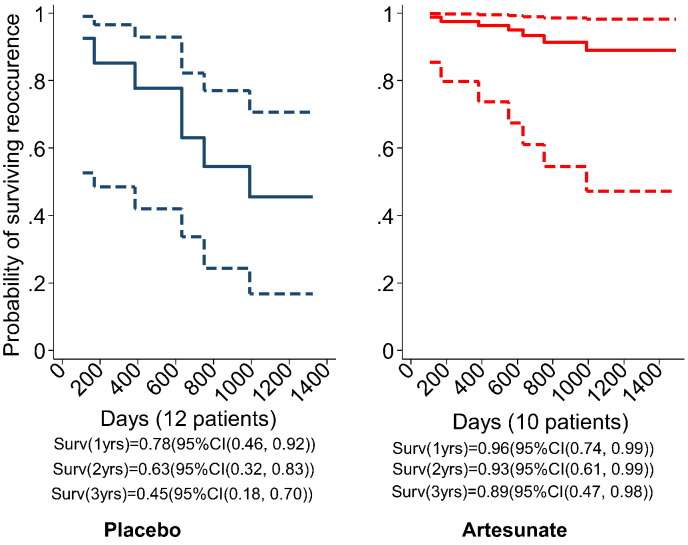
Survival recurrence curves predicted by Cox proportional hazards model. Patient CRC21 was assumed to be missing completely at random (please see Supplementary Table 1 for a full sensitivity analysis). In the placebo group 2 patients died within a year (108, 170) days leaving 10 (83%) in the study, another 2 within the next year (383, 663 days) leaving 8 (66%) in the study and the other two died within the third year of the follow up (749 and 990, respectively) leaving 50% patients beyond the third year. The only death in the artesunate group happened after 552 days leaving 9 patients (90%) surviving beyond the third year. These crude estimates support the estimates from the data above.

During a median follow up of 42 months, there were 6 recurrences in the placebo group and 1 recurrence in an artesunate recipient. Fig. 4 illustrates results from a Cox's proportional hazards model. The hazard ratio of first disease recurrence is 0.16 (95% CI (0.02, 1.3)) in the artesunate group compared with placebo. The survival beyond 2 years in the artesunate group is estimated at 91% (95% CI (54%, 98%)) whilst surviving the first recurrence in the placebo group is only 57%(95% CI (28%, 78%)). A full sensitivity analysis for patient CRC21 is given in Supplementary Table 1 and suggests that if this patient was in the artesunate group without recurrence at 3 years, then a p-value for an artesunate effect on survival would be p = 0.07 (95% CI 0.02, 1.21).

#### 3.4.3 CEA Levels

Six artesunate and 4 placebo recipients had CEA levels measured before and after trial medication (and before resection, classified as reduced, stable or increased). No patients with artesunate had increased CEA levels whereas 3 patients in the placebo group had increased values (p = 0.03, Fisher's exact test).

### 3.5 Adverse Events

**Fig. 5:**
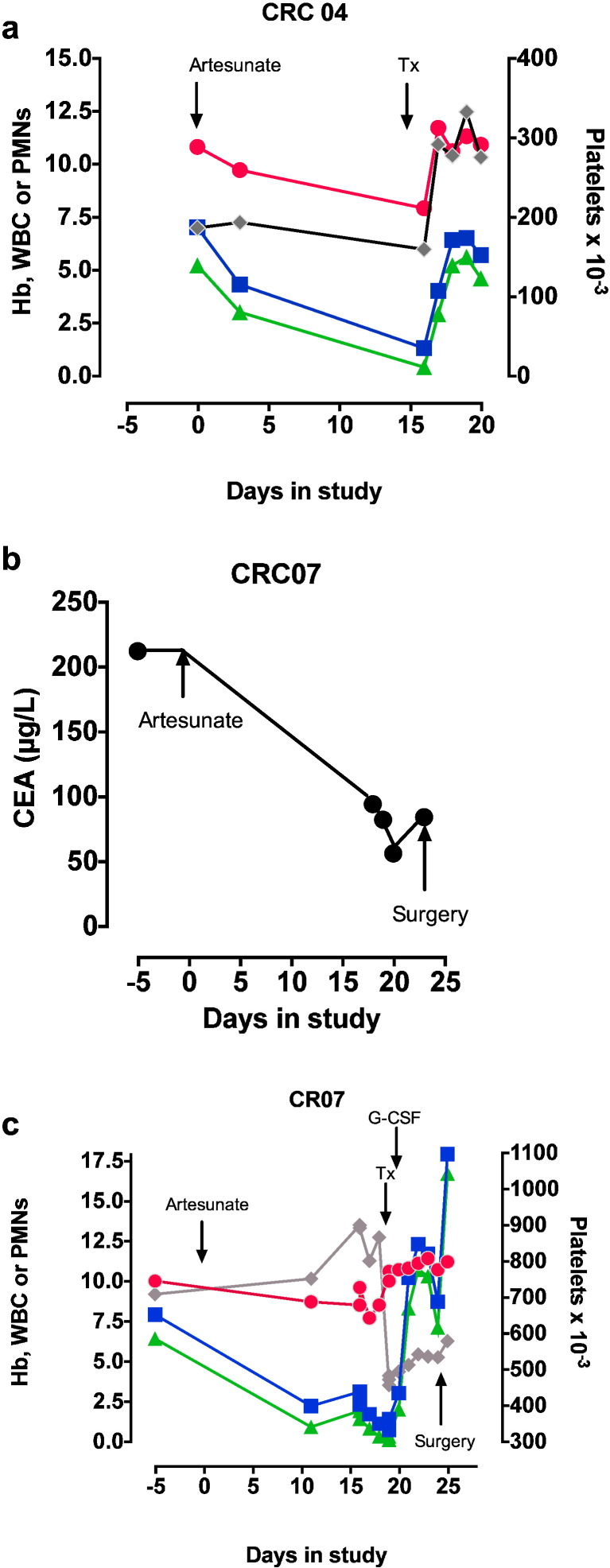
Adverse events. a. Patient CRC 04's haemoglobin (filled red circles, g/dl), total white cell count (filled blue squares, × 10^− 9^/L), neutrophil count (green triangles, × 10^− 9^/L) and platelet count (grey diamonds, x) are shown from the start of the study (Day 0). b. CRC 07's serum carcinoembryonic antigen levels are shown from the start of the study (Day 0). c. CRC 07's haematological results are shown from the start of the study (Day 0) with symbols as in a. Tx is transfusion of red cells.

Six patients (26% for the ITT population) had adverse events (2 severe, Table 3 and Supplementary Table 1). Two adverse events possibly related to study drug are described in detail. In the remaining 4 cases, 2 complications (anastomotic leaks after surgery) were considered unlikely to be related to artesunate, and one case of iron-deficiency anaemia (with no neutropaenia) was attributed to underlying disease. There was one report of nausea. Detailed descriptions of 2 cases of neutropaenia are given below and illustrated in Fig. 5.

CRC04: An 81 yo 51 kg female presented with anaemia and a change in bowel habit, and was discovered to have a large, annular ascending colon, polypoidal, carcinoma that was not producing obstruction on colonoscopy. She was randomised to receive artesunate. There was no evidence of metastatic spread on staging scans (CEA = 3 μg/L). Her mid-treatment review was unremarkable. She returned for surgery and was found to be anaemic and neutropenic (Fig. 4a). She was transfused, making this a Grade 3 adverse event according to CTCAE criteria (v4.0; http://evs.nci.nih.gov/ftp1/CTCAE/CTCAE_4.03_2010-06-14_QuickReference_5x7.pdf). Her neutropenia recovered the following day.

She underwent a laparoscopic right-sided hemicolectomy with an uneventful recovery. She was offered post-operative chemotherapy in light of her moderately differentiated pT3, Dukes' stage B adenocarcinoma, which she declined. This was defined as a non-severe adverse event as there was no prolongation in hospitalization.

CRC07: A 79 yo 50 kg lady presented with anaemia, rectal bleeding and a change in bowel habit in the preceding few months. She had a history of endometrial carcinoma and underwent a total abdominal hysterectomy and bilateral salpingo-oophorectomy followed by radiotherapy 11 years before.

Colonoscopy confirmed an adenocarcinoma with impassable stricture at the splenic flexure. Staging CT scan of the chest, abdomen and pelvis excluded metastasis. She was randomised to receive artesunate. Her CEA was 212 μg/L but fell steeply following artesunate (to a nadir of 56 μg/L, Fig. 4b) and no other intervention. She had a persistent thrombocytosis, but she developed anaemia and leucopenia, which was noted on the day of her planned surgery (Fig. 4c). Her pre-surgical screening carried out 5 days before surgery also showed anaemia and leucopenia. Her surgery was delayed and after appropriate expert consultation and bone marrow examination her anaemia was treated with blood transfusions and her persistent neutropenia with G-CSF, making this also a Grade 3 adverse event according to CTCAE criteria.

Bone marrow aspiration showed normal erythropoiesis, with dysplastic granulopoiesis and maturation arrest. Some myeloid precursors were vacuolated with nucleocytoplasmic asynchrony, but without excess myeloblasts. Megakaryocytes were plentiful. There was a slight increase in plasma cells. Findings were consistent with drug induced myelosuppression.

The neutrophil count rose after 2 days of G-CSF, which was stopped. The patient underwent a left hemi-colectomy with *en bloc* resection of a small segment of involved small bowel and primary colonic and small bowel anastamosis 11 days after artesunate, without any post-operative complications, and a fall in platelet count (Fig. 4c). The adenocarcinoma was staged as a Dukes' C1 and T4abN2M0. The tumour was found to be poorly differentiated signet ring type adenocarcinoma with extramural vascular invasion. She was advised to have adjuvant chemotherapy as the risk of recurrence was ~ 50%. She declined and opted for surveillance alone. After 3 years of follow up she is confirmed to be symptom and disease free and continues to lead an independent life.

## 4 Discussion

This is the first randomised, double blind study to test the anti-CRC properties of oral artesunate. Escape from apoptosis is a hallmark of tumour cells (Fiandalo and Kyprianou, 2012), with higher apoptotic indices being associated with more aggressive CRCs (Alcaide et al., 2013). The pre-defined primary endpoint (proportion of patients with > 7% Tunel positive staining of tumour cells) after artesunate treatment was not informative, perhaps because an unexpectedly high proportion (55%) of placebo recipients exceeded the pre-defined threshold. Nevertheless, several secondary endpoints have given encouraging results, despite limitations of a small study size and an inherent variability in quantitating immunohistochemical markers.

Artesunate has a very high probability (0.97, calculated with an informative prior in Bayesian analysis, Fig. 2b) of effect on Ki67 staining of tumour cells. This is consistent with a high probability of artesunate effect on Ki67 staining of fibroblasts (0.84; Table 2). Ki67 is a marker of tumour cell proliferation whose upregulation is associated with a poorer prognosis in colorectal cancer. Other markers of tumour biology were also affected by artesunate, although with lower probabilities (for example, 0.79 probability for increased CD31 expression). In one case (Fig. 4b) there was a ~ 75% fall in circulating CEA levels after 2 weeks of artesunate treatment alone.

The recurrence-free survival probability was also higher after artesunate compared with placebo (at 3 years 0.89 compared with 0.5; Fig. 3) although confidence intervals for these estimates overlap (HR 0.16, p = 0.091, Supplementary Table 1) because of the small numbers of patients and therefore events included in this study. Till this analysis, there have been no deaths in artesunate recipients (despite some patients having relatively poor prognosis), and 3 deaths in placebo recipients.

Two patients who were at the lower weight limit for inclusion in this study (50 kg, giving an effective dose of 4 mg/kg of artesunate/day) developed leucopenia (Fig. 4). In one case this reversed shortly after stopping artesunate, whereas in the other G-CSF may have hastened recovery. Bone-marrow examination suggested a toxic effect of artesunate. These findings are consistent with the recent observations in malaria of a dose-dependent neutropenia with artesunate (> 4 mg/kg) (Bethell et al., 2010), although bone marrow examinations have not been carried out before. We instituted mid-treatment monitoring for neutropenia after observations on malaria but did not note this complication in other patients. Artesunate associated leucopenia may be dose-dependent in cancer patients as it is in malaria, and although delayed haemolysis has been observed after artemisinin use (Rolling et al., 2012, Rolling et al., 2014) it was not a complication in our patients. In future studies, it may be safer to restrict daily dose of artesunate to < 4 mg/kg and to monitor for haematological complications. A recent publication on an artesunate dose-finding study in metastatic breast cancer disease suggests that 200 mg once a day can be tolerated for up to 3 weeks (Ericsson et al., 2014).

Liver recurrence was commonest in our patients, followed by peritoneal and ovarian sites, suggesting that seeding is mainly haematogenous and trans-peritoneal. As patients had clear circumferential and longitudinal margins at surgery, and detectable metastases were not identified at randomisation, it is likely that micrometastases spread through vascular invasion (VI) caused recurrence. Previous experience suggests that VI predicts decreased survival in CRC (Ganapathi et al., 2011, Liang et al., 2007, Talbot et al., 1980, Betge et al., 2012). Patients with cryptic dissemination of CRC may benefit from systemic neo-adjuvant therapy and artesunate may be particularly suitable because it does not usually delay surgery. It also reduces liver metastases in an animal model (Li et al., 2007).

These observations provide critical information for the design of further studies. In assessing its neo-adjuvant properties, we have also examined artesunate's mechanisms of action in human CRC. Artesunate does not restore apoptosis in tumour cells in our study, but rather decreases the expression of a Ki67. Ki67 is also an important marker of prognosis in CRC unlike CD31, which is increased in expression. These findings are consistent with uncontrolled observations made in cervical cancer (Jansen et al., 2011), where decreased Ki67 staining was also observed, although decreased CD31 staining of blood vessels in that study contrasts with our observations. Detailed laboratory observations on anti-cancer mechanisms of artesunate such as on proliferation (Efferth et al., 2003, Efferth et al., 2007, Yeung et al., 2013) and expression of tumour cell markers (Konkimalla et al., 2009, Efferth et al., 2004, Konkimalla and Efferth, 2010) (including for angiogenesis) can now be interpreted in light of *in vivo* observations. Larger clinical studies with artesunate that aim to provide well tolerated and convenient anticancer regimens should be implemented, and may provide an intervention where none is currently available, as well as synergistic benefits with current regimens.

## Figures and Tables

**Table 1:** Baseline demographic, clinical and laboratory characteristics.

	Artesunate N = 12 Mean (SD)	Placebo n = 11 Mean (SD)	Numbers^a^ (artesunate/placebo)
*Demographics*			3
Age (y)	69 (11)	66 (14)	12/10
Gender (%, F)	0.58	0.64	
Ethnicity (% Caucasians)	0.83	0.82	
Height (m)	1.70 (0.12)	1.62 (0.08)	10/9
Weight (kg)	74 (16.7)	75 (66, 85)	11/10

*Biochemistry*			
Sodium (mmol/L)	140 (1)^a^	139 (2.28)	11/11
Albumin (g/L)	38 (5.7)	36 (5.7)	11/11
ALT (U/L)	23 (8)	24 (10)^a^	11/11
Bilirubin (μmol/L)	9 (3)	8 (3)	9/11
Creatinine (μmol/L)	73 (23)	65 (15)	9/11
Urea (mmol/L)	5 (2)	5 (1)	9/11

*Haematology*			
Haemoglobin (g/dL)	11.5 (2)	12.2 (2)^a^	9/11
White Cell count (/L)	5.3 (2.8)	6.7 (2)	9/11
Platelet Count (/L)	370000 (20000)^a^	31300 (78000)	8/11

*Dukes' stage*			
A	2	2	
B	5	3	
C1	2	6	
C2	1	1	

a Numbers of patients contributing to each value are indicated in the last column.

**Table 2:** Predicted “grand means” of immunohistochemistry results. Results are presented on a linear scale by treatment groups with their 95% credible intervals following a Bayesian analysis which takes into account the variability within individual measurements as well as that of between different individuals in the cluster. The probability of an effect is the probability that the difference between the grand means in the two groups (artesunate–placebo) is greater than 0 (and is not a p-value). *For Ki67 all corresponding estimates with informative priors are presented in after sensitivity analysis, with skeptical priors presented first followed by informative priors. Results for some epithelial stains were correlated with each other in placebo recipients (EGFR and c-MYC; r = 0.664; 0.026) and for artesunate recipients (EGFR and p53, r = 0.68; p = 0.04, and Tunel and p53 r = 0.87; p = 0.0025).

Marker	Artesunate		Placebo		P of an effect	Difference or change	Sensitivity analysis (prior)
Mean	95% CrI	Mean	95% CrI
*Epithelial*							
cMYC	45	(27, 53)	43	(27, 64)	0.59	2.6 (− 22, 26)	
CD31	8	(3,12)	5	(2,9)	0.79	2.6 (− 4, 9)	
EGFR	32	(12, 58)	27	(9, 45)	0.66	5.4 (− 24, 32)	
p53	25	(5, 45)	18	(3,35)	0.72	7 (− 20, 30)	
Tunel	18	(4,32)	20	(7,32)	0.4	− 2 (− 20, 17)	
VEGF	38	(21, 54)	43	(40, 75)	0.45	− 5 (− 28, 17)	
Ki67*	33	(13, 52)	49	(31, 66)	0.11	− 16 (− 42, 10)	N (0, 1000) Non-informative
	34	(14, 53)	48	(31, 65)	0.13	− 15 (− 40, 10)	N (0, 100) Vaguely informative
	38	(22, 54)	44	(29, 59)	0.23	− 6 (− 22, 10.1)	N (0, 10) Informative
	32	(12, 51)	49	(32, 66)	0.08	− 18 (− 42, 8)	N (− 15, 1000) Non-informative
	32	(13, 51)	49	(32, 66)	0.08	− 18 (− 42, 8)	N (− 15, 100) Vaguely informative
	32	(17, 49)	48	(33, 63)	0.03	− 15 (− 31, 0)	N (− 15, 10) Informative

*Fibroblast*							
c-MYC	7	(− 7, 21)	19	(7,32)	0.10	− 12 (− 30, 7)	
CD31	13	(− 2, 26)	11	(0, 23)	0.58	1 (− 17, 19)	
EGFR	0.1	(− 0.5, 0.7)	0.4	(0, 0.9)	0.21	− 0.3 (− 1, 1)	
Ki67	5	(− 3, 12)	10	(3,16)	0.16	− 5 (− 15, 5)	
p53	0.7	(− 0.8, 2.1)	0.9	(− 0.3, 2)	0.40	− 0.2 (− 2.1, 1.2)	
Tunel	3	(0, 5)	4	(1,6)	0.31	0.8 (− 4, 3)	
VEGF	0.2	(− 0.02, 0.5)	0.2	(0.1, 0.3)	0.52	0.02 (− 0.3–0.3)	

**Table 3:** Adverse events. For treatment allocations, please see Fig. 2a.

Study number	Event	Due to pre-existing illness	Related to study drug	Serious?	Study treatment	Outcome
CRC 004	Neutropaenia and anaemia	No	Possibly	N		Resolved
CRC 007	Neutropaenia and anaemia	No	Possibly	Y	Stopped	Resolved
CRC 017	Nausea, but no vomiting	No	Possibly	N	Continued unchanged	Resolved
CRC 018	Anastomotic leak	No	Not related	Y		Resolved
CRC 019	Anastomotic leak	No	Unlikely	Y	Stopped	Resolved
CRC 022	Anaemia	Yes	Unlikely	N	Continued unchanged	Resolved
