# Supplementary material for: A Randomised, Double Blind, Placebo-Controlled Pilot Study of Oral Artesunate Therapy for Colorectal Cancer
Source: eBioMedicine. 2014 Nov 15;2(1):82–90. doi: 10.1016/j.ebiom.2014.11.010 (PMC4484515; doi:10.1016/j.ebiom.2014.11.010)
Supplement: Supplementary file 1 — Supplementary Material 1. [file mmc1.docx]

**Supplementary Table 1**

| Sensitivity analysis for CRC21 | Hazard Ratio:  Artesunate vs. Placebo | p-value | 95% CI |
| --- | --- | --- | --- |
|  |  |  |  |
| Original - CRC21 MCAR | 0.16 | 0.091 | (.019, 1.34) |
| Artesunate, no recurrence | 0.146 | 0.075 | (.017, 1.21) |
| Artesunate, recurrence 1 y | 0.31 | 0.16 | (.063, 1.55) |
| Artesunate, recurrence 2 y | 0.30 | 0.14 | (.060, 1.47) |
| Artesunate, recurrence 3 y | 0.27 | 0.111 | (.054, 1.35) |
| Placebo, no recurrence | 0.18 | 0.111 | (.021, 1.49) |
| Placebo, recurrence 1 y | 0.14 | 0.069 | (.018, 1.16) |
| Placebo, recurrence 2 y | 0.15 | 0.073 | (.018, 1.19) |
| Placebo, recurrence 3 y | 0.14 | 0.070 | (.018, 1.17) |

Survival analysis for the hazard ratio of recurrence for artesunate vs. placebo groups. The sensitivity analysis is carried with regards to patient CRC21 who has been tested as being assigned to each group, and also for the assumption of recurrence. This patient was diagnosed on 12^th^ May 2011 and has been lost to follow up.

**Supplementary Methods**

*Changes to trial design*

After original submission, the research protocol was amended at the following times and for the reasons given: v2.2 25/3/08 (non-acceptance of v2.1 by MHRA); v2.3 9/9/08 (change to QP release details on CTA and protocol); v2.4 16/3/09 (additional blood sampling to include peripheral blood mononuclear cells); v2.5 11/5/10 (IMP change of strength and manufacturer, increased age range for inclusion from 80 y to 90 y; inclusion of surgically treatable CRC not requiring neo-adjuvant therapy and not only those who are potentially curable, and enhanced monitoring for AEs by clinical and haematological checks after 1 week of drug/placebo); v2.6 7/9/10 (minor change in number of tablets quoted for treatment).

*Interim analyses and stopping guidelines*. One interim analysis was planned for futility when the first 10 patients completed the study, and was performed accordingly. These cases were unblinded for the statistician only, and the recommendation was that recruitment should continue. Interim analysis used different software to that used to evaluate slides for the final analysis, which was still carried out in a blinded fashion.

*Immunohistochemistry*

Formalin-fixed, and paraffin-embedded sections were deparaffinated (2×2 min xylol) and rehydrated. For antigen retrieval, sections were submerged in Target Retrieval Solution (Thermo Fisher Scientific) for 20 minutes at 95-99°C. Afterwards, slides were allowed to cool to room temperature and washed (PBS, pH 4; 10 min). Endogenous peroxidase activity was blocked (in 3% H_2_O_2_, 10 min at room temperature). After rinsing (5 m in PBS) blocking was by Ultra Vision Block (5 m; Thermo Fisher Scientific).

Slides were incubated, humidified and overnight at 4°C with primary antibody: Ki-67 (MIB-1, dilution 1:200, DAKO GmbH, Hamburg, Germany), p53 (318-6-11, dilution: 1:200, DAKO), EGFR (EP38Y, dilution 1:50, Thermo Scientific), c-MYC (9E10.3, dilution 1:50, Thermo Scientific), CD31 (JCI70A, dilution 1:50, Thermo Scientific) and VEGF (VG1, dilution 1:150, Thermo Scientific). After rinsing as before, Primary Antibody Amplifier Quanto (Thermo Fisher Scientific) was applied (10 m at room temperature). Slides were washed as before and then HRP Polymer Quanto (Thermo Fisher Scientific) was applied for 10 min and a wash step (5 min) followed. Diaminobenzidine (30 µL, DAB) Quanto chromogen (Thermo Fisher Scientific) was mixed with DAB Quanto substrate (1 mL) and applied to the slides for 5 min. For the detection of apoptotic cells, the Apotaq® TUNEL apoptosis detection kit (Merck Millipore, Darmstadt, Germany) was used. After washing as before, the tissues were counterstained in haemalaun solution (Merck KGaA, Darmstadt, Germany) and rinsed in PBS for 5 min, followed by running tap water (10 min). Tissue sections were dehydrated (2×1 min 70% ethanol, 2×1 min 96% ethanol, 2×1 min 100% ethanol, 2×5 min xylol, 1×2 min xylol) and embedded using Entellan (Merck).
