# Supplementary material for: A Randomised, Double Blind, Placebo-Controlled Pilot Study of Oral Artesunate Therapy for Colorectal Cancer
Source: eBioMedicine. 2014 Nov 15;2(1):82–90. doi: 10.1016/j.ebiom.2014.11.010 (PMC4484515; doi:10.1016/j.ebiom.2014.11.010)
Supplement: Supplementary file 2 — Supplementary Material 2. [file mmc2.doc]

**
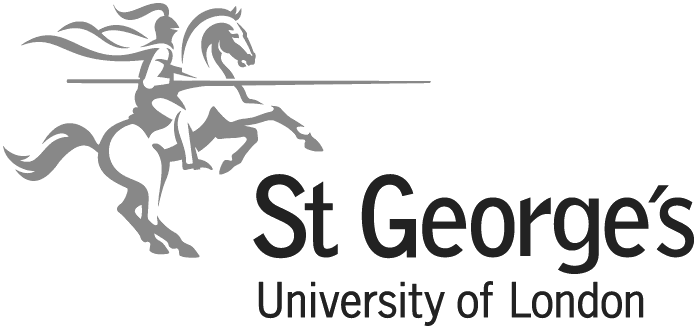
**

**Phase I placebo-controlled, randomized, double-blind tolerability and efficacy study of oral artesunate in patients with colorectal carcinoma**

**Principal Investigator: Professor Devinder Kumar**

**Co-Principal Investigator: Professor**

**Study sponsor: St George’s, University of London**

**Version 2.5**

**11^th^. May 2010**

# Contents

Contents [2](#__RefHeading___Toc58666077)

Introduction [4](#__RefHeading___Toc58666078)

Background [4](#__RefHeading___Toc58666079)

Mechanisms of action [6](#__RefHeading___Toc58666080)

Summary [6](#__RefHeading___Toc58666081)

Study Objectives [7](#__RefHeading___Toc58666082)

Study Design [8](#__RefHeading___Toc58666083)

Selection of Patients [9](#__RefHeading___Toc58666084)

Inclusion criteria [9](#__RefHeading___Toc58666085)

Exclusion criteria [9](#__RefHeading___Toc58666086)

Recruitment [9](#__RefHeading___Toc58666087)

Study Medication [11](#__RefHeading___Toc58666088)

Artesunate and placebo [11](#__RefHeading___Toc58666089)

Drug dosing [11](#__RefHeading___Toc58666090)

Packaging and storage conditions [11](#__RefHeading___Toc58666091)

Drug interactions with artesunate [12](#__RefHeading___Toc58666092)

Adverse events associated with artesunate [12](#__RefHeading___Toc58666093)

Pregnancy [13](#__RefHeading___Toc58666094)

Assessment of medication compliance [13](#__RefHeading___Toc58666095)

Patient Safety [14](#__RefHeading___Toc58666096)

Definitions of adverse event and serious adverse event [14](#__RefHeading___Toc58666097)

Obligation of the investigator regarding safety reporting [15](#__RefHeading___Toc58666098)

Pregnancy [16](#__RefHeading___Toc58666099)

Obligations of Sponsor [16](#__RefHeading___Toc58666100)

List of criteria for discontinuation of study medication [16](#__RefHeading___Toc58666101)

Handling of patients after treatment discontinuation [17](#__RefHeading___Toc58666102)

Pregnancy [17](#__RefHeading___Toc58666103)

List of criteria for premature discontinuation of the study [17](#__RefHeading___Toc58666104)

Study Procedures [18](#__RefHeading___Toc58666105)

Visit schedule [18](#__RefHeading___Toc58666106)

Treatment group assignation [19](#__RefHeading___Toc58666107)

Data collection [19](#__RefHeading___Toc58666108)

Outcome Measures [21](#__RefHeading___Toc58666109)

Primary outcome measure - efficacy [21](#__RefHeading___Toc58666110)

Secondary outcome measures - tolerability [22](#__RefHeading___Toc58666111)

Other histological assessments [22](#__RefHeading___Toc58666112)

Interim analysis [23](#__RefHeading___Toc58666113)

Blinding [23](#__RefHeading___Toc58666114)

Statistics [23](#__RefHeading___Toc58666115)

Ethical and regulatory standards [24](#__RefHeading___Toc58666116)

Miscellaneous Considerations [25](#__RefHeading___Toc58666117)

Financing and Insurance [25](#__RefHeading___Toc58666118)

Insurance Compensation [25](#__RefHeading___Toc58666119)

Publication Policy [25](#__RefHeading___Toc58666120)

Study Personnel [26](#__RefHeading___Toc58666121)

Scientific Advisory Panel [26](#__RefHeading___Toc58666122)

Research Fellows [26](#__RefHeading___Toc58666123)

Clinical Trials Pharmacists [27](#__RefHeading___Toc58666124)

Data Monitoring Committee [27](#__RefHeading___Toc58666125)

Sponsor’s representative [28](#__RefHeading___Toc58666126)

References [29](#__RefHeading___Toc58666127)

Appendix 1: Study design [31](#__RefHeading___Toc58666128)

Appendix 2: Sample Case Record Form [32](#__RefHeading___Toc58666129)

Appendix 3: Patient Information Sheet [42](#__RefHeading___Toc58666140)

# Introduction

The overall aim of this study is to assess the tolerability and anti-cancer properties of oral artesunate (an established antimalarial agent) for colorectal cancer (a new indication).

## Background

Colorectal cancer (CRC) is the third commonest cancer in industrialised countries, with over 1 million individuals diagnosed each year [1]. Early diagnosis and surgical treatment can be curative. However, up to half of the patients with CRC present with advanced stages of disease and require adjuvant therapy, where the chances of cure are correspondingly diminished.

Current adjuvant therapies now include expensive biologically targeted agents such as the humanised monoclonal anti-VEGF antibody bevacizumab given with combination anti-cancer chemotherapeutic regimens that contain 5-fluorouracil with oxaliplatin (FOLFOX) or with irenotecan (FOLFIRI) [2]. Despite demonstrated survival benefits with bevacizumab used in combination with existing chemotherapeutic regimens, it is not yet approved by NICE, and use has to be agreed case-by-case (see: <http://news.bbc.co.uk/1/hi/england/london/6904342.stm>). As for other cancers where biological agents are proving to be useful, their expense can limit deployment. Most patients with advanced disease will not survive more than 5 years after diagnosis. There is therefore an urgent need to improve survival outcomes in patients with metastasised CRC using affordable interventions, if possible.

Artesunate (Figure 1) is one of the most widely used antimalarial compounds in the world. It was discovered by Chinese scientists in the early 1970s, and since then has proved itself (together with other derivatives in this class of artemisinin compounds, such as artemether and dihydroartemisinin) effective and safe when used in the treatment of tens of millions of adults and children [3-5].

**Figure 1.** Artesunate (artesunic acid). The chemical structure shows an endoperoxide bridge (red) and a 3^rd^. oxygen (purple) that together making up a trioxolane moiety essential for antimalarial activity.

**O**

**O**

**O**

**O**

**O**

**H**

**H**

**O**

**O**

**O**

**H**

**5**

α

Efferth first showed that artesunate has anti-cancer activity when tested against leukaemia cells [6] and subsequently dozens of cell lines [7]. Highest anti-cancer activity was against leukaemia and CRC cell lines, although this class of compound has activity against a large number of difficult-to-treat unrelated cancers, including ovarian cancers, melanomas and sarcomas (reviewed in [8]).

These *in vitro* studies are supplemented by studies in animal models that confirm anti-cancer efficacy, including a recent publication describing mechanism of action and efficacy of artesunate in a colorectal carcinoma model in nude mice. Shrinkage of the primary tumour as well as delay in development of liver metastases was attributed to inhibition of the Wnt/β-catenin pathway [9].

Published studies of anti-cancer efficacy of artesunate or artemether monotherapy are limited to case reports that include: metastatic uveal melanoma (2 cases) [10], laryngeal squamous cell carcinoma [11] and pituitary macroadenoma [12].

There is also unpublished evidence from Dr. H. Jansen (collaborator on this application, who has consented to this experience being presented) of use of oral artesunate after colonoscopically confirmed CRC before surgery. As this experience informs the current trial design, it is reported here:

*Case report*: In December 2006, a 60 year old medically qualified pharmaceutical chemist was diagnosed at colonoscopy with Stage IIIB CRC with 7 lymph nodes noted on CT scanning. Sigmoidoscopic sizing of the adenocarcinoma was 12 x 5 cm. He began taking 200 mg of oral artesunate daily, and after 24 hours developed mild ‘flu-like symptoms with muscle pains, slight fever and some weakness. These symptoms resolved after 3 days, and artesunate was continued for a total of 11 days. The tumour was resected and found to be 5 x 4.5 cm in size. Histology of the tumour showed multiple areas of apoptosis and necrosis, and only 2 of the 7 lymph nodes identified at biopsy contained evidence of carcinoma. Since operation (10 months) the patient has continued to take artesunate (200 mg, daily for 2 weeks with one week off artesunate), and has completed 10 cycles of conventional treatment with 5-fluorouracil.

## Mechanisms of action

The mechanisms of action of artesunate as an antimalarial and its anti-cancer properties are likely to differ (reviewed [13] [8] [14]). Whatever the molecular basis for anti-cancer effects of artesunate, pro-apoptotic [6, 15] and anti-angiogenic properties [16] are observed in diverse experimental models of cancer, including CRC [9].

## Summary

Artesunate is a safe and effective antimalarial that has not yet been examined systematically for its anticancer properties, despite there being a compelling scientific rationale for doing so. At St. George’s, there is world-class expertise in the use of artesunate (all aspects from mechanisms of action [17] [18], up to Phase IV clinical studies with the drug [19] [20] [21]) as well as in the pathophysiology and multidisciplinary management of CRC [22] [23, 24]. In addition, we have developed collaborations with the first group to have defined anti-cancer properties of artemisinins ([7, 8]).

# Study Objectives

The primary objective of the study is to determine the anticancer effect of oral artesunate in colorectal adenocarcinoma defined as the proportion of malignant cells undergoing apoptosis.

Secondary outcome measures are to establish the tolerability of oral artesunate in colorectal cancer and the effects of artesunate on tumour markers and other clinical endpoints.

# Study Design

This will be a Phase I randomised placebo-controlled double-blind trial of artesunate given orally to patients with histologically confirmed CRC who will receive treatment with curative intent. The study will involve 22 patients randomly assigned to receive artesunate or placebo. The overall study duration will be 14 days. An interim efficacy analysis is planned after the first ten patients have completed the study.

A Data Monitoring Committee (DMC) will supervise the study. The primary role of the DMC is to protect the safety of the patients participating in the study by reviewing the safety information. Clear criteria for terminating the study prematurely are given. The DMC is composed of three individuals, an individual with expertise in the conduct of drug trials involving artesunate, a statistician and a colorectal surgeon with a specialist knowledge of the management of colorectal cancer.

# Selection of Patients

## Inclusion criteria

- Aged 21 – 90 years
- With biopsy confirmed single primary site colorectal adenocarcinoma
- With all stages (defined according to conventional criteria, summarised in [1]) that are amenable to surgical treatment and do not require neoadjuvant treatment
- With planned resection
- With written, informed consent

## Exclusion criteria

- Contraindication to use of artesunate due to hypersensitivity
- Pregnancy (of any stage)
- History of hearing or balance problems
- Immunosuppression or concomitant medication known to interact with artesunate (see below)
- Weight < 50 kg or > 100 kg
- Severe anaemia (haemoglobin < 8 g/dl)
- Other planned intervention, apart from standard of care
- Inability to give informed consent
- Inability or unwillingness to take effective contraception in women of child-bearing age
- Chronic kidney disease of NKF D/QOFI stage 3 or above (eGFR <60ml/min)
- Bilirubin > 2x upper limit of normal in the absence of haemolysis, or known chronic liver disease

## Recruitment

Potential subjects will be identified from lists of patients attending the surgical oncology clinic or from lists of patients discussed at the weekly Lower Gastrointestinal Cancer Multidisciplinary Meeting. Patients who have received histological confirmation of their diagnosis and are planned for curative resection, will be recruited by a Surgical or Medical Research Fellow, who has been trained in clinical research to standards of Good Clinical Practice. Patients will be given an information sheet (Appendix 3, May 2010, version 4) regarding the trial and procedures. They will have an opportunity to question the researchers and time to come to a decision about whether or not to take part in the study. There will be no delay in surgery if patients are entered into this study, nor any other change in clinical management, and the 62 day rule will be strictly adhered to.

# Study Medication

## Artesunate and placebo

Artesunate (Arinate® 100mg) and matching placebo tablets are manufactured under Licence according to cGMP by Dafra Pharma (Belgium). Quality Control of study medication is assured by Dafra Pharma in accordance with the apropriate regulatory framework. This will be the only material contribution of Dafra to the trial. **Study medication will be packaged, labelled and certified by B&C CliniPack (Belgium) and will be in pack sizes of 30 x 100 mg.** Study medication will be imported, stored and dispensed by the Pharmacy at St George’s Healthcare NHS Trust.

## Drug dosing

The dose of artesunate for the study will be 200mg od for fourteen days. There are no data on the most appropriate dose of artesunate in cancer in humans. This dose has been chosen by a consensus among experts in the field of artesunate.

The daily dose of artesunate used in combination treatments to treat uncomplicated *Plasmodium falciparum* malaria is 4 mg/kg, given over three days. When given as monotherapy for uncomplicated falciparum malaria the total dose is approximately 20mg/kg, over seven to ten days. The total dose of artesunate for a patient in this study will be between 28 mg/kg and 56 mg/kg, depending on the weight of the subject (ranging between 50-100 kg).

## Packaging and storage conditions

Study medication will be provided in blister packs. One patient box will provide 14 doses, sufficient for the duration of the study. The content of the labelling will be in accordance with local regulatory specifications and requirements.

Arinate® has a shelf life of 3 years if stored at room temperature.

## Drug interactions with artesunate

There are no reports of negative drug interactions with artesunate to date. The manufacturers of artemesinins available in the UK advise against concomitant use with:

- Anti-arrhythmics: procainamide or quinidine
- Antibacterials: macrolides and quinolones
- Antidepressants
- Antifungals: imidazoles and triazoles
- Antipsychotics

Patients who are taking these medications will not be recruited. If such treatment is required on medical grounds, and cannot wait until the end of the study period, the trial medication will be withdrawn and the reason documented by the investigators.

## Adverse events associated with artesunate

Artesunate is a safe drug that has been taken by millions of people worldwide. There are no data on adverse events associated with artesunate in the treatment of cancer in humans. Data are taken from studies in malaria. Serious side-effects are extremely rare. Adverse events include:

- Gastrointestinal: nausea, diarrhoea, abdominal cramps, increased transaminase levels
- Haematological: transient reduction in reticulocyte count and possibly white cell count (see Adverse event reports from this study)
- Neurotoxicity: ototoxicity and neurotoxicity has been reported in animal models when extremely high doses of artesunate (100mg/kg) have been administered intravenously.

## Pregnancy

Artesunate is teratogenic in animal models and is contraindicated in pregnancy in humans. Pregnant women will not be recruited and subjects will be required to use effective methods of contraception during the study.

## Assessment of medication compliance

An assessment of medication compliance will be made by the investigators at the end of the treatment course based upon verbal answers given to direct questioning and a count of untaken medication. Any unused medication will be returned to the Pharmacy at St George’s Healthcare NHS Trust for disposal in a self-addressed stamped envelope provided to the patient.

# Patient Safety

## Definitions of adverse event and serious adverse event

All events occurring whilst participants are taking the study medication will be recorded on the case record form and managed appropriately in accordance with local and national regulations.

An adverse event (AE) is defined as any untoward medical occurrence in a patient administered a pharmaceutical product and which does not necessarily have to have a causal relationship with this treatment. A priori, efficacy endpoints will not be considered as AEs except if, because of the course or severity or any other features of such events, the investigator, according to his/her best medical judgement, considers these events as exceptional in this medical condition. All AEs will be classified according to the Common Terminology Criteria for Adverse Events v3.0 of the Cancer Therapy Evaluation program.

A serious adverse event (SAE) is any untoward medical occurrence that:

- Results in death or;
- Is life threatening or;
- Requires inpatient hospitalisation or prolongation of existing hospitalisation or;
- Results in persistent or significant disability/incapacity or;
- Is a congenital anomaly/birth event or;
- Is a medically important event

Medical and scientific judgement will be exercised in deciding whether expedited reporting is appropriate in other situations, such as important medical events that may not be immediately life-threatening or result in death or hospitalisation but may jeopardise the patient or may require intervention to prevent one of the other outcomes listed in the definition above.

## Obligation of the investigator regarding safety reporting

All AEs, regardless of seriousness or relationship to the study medication are to be recorded in the Case Record Form. Whenever possible, symptoms will be grouped as a single syndrome or diagnosis. The investigator will specify the date of onset, intensity, action taken with respect to study medication, corrective treatment/therapy given, outcome and his/her opinion as to whether there is a reasonable possibility that the AE was caused by the study medication.

In the case of a SAE the investigator must immediately:

- Ensure appropriate medical care of the study participant and follow-up until clinical recovery is complete
- Inform, in writing, the DMC including a signed and dated copy of the case record form
- Inform the study Sponsor using the appropriate local form (available at <https://intranet.sgul.ac.uk/admin/research/clinic/index.php>)
- In the case of any SAE brought to the attention of the investigator at any time after the cessation of the study medication and considered by him/her to be caused by the study medication with a reasonable probability, this should be reported to the DMC

In the case of an AE, when knowledge of the identity of the study medication is essential for treating the patient, the code for randomisation will be broken. Wherever possible this should be initiated by the DMC. In the event of an emergency an unblinding procedure may be authorised by the investigator. If the blind is broken, the investigator will document the time, date and reason for code breaking in the case record form.

**Laboratory test monitoring**

Abnormalities of laboratory values developing during the study period, or worsening of pre-existing abnormal values, during the duration of the study period will be recorded as AEs.

## Pregnancy

In the unlikely event of pregnancy occurring the study medication will be stopped and the sponsor informed. Follow-up of the pregnancy will be mandatory in all cases until the outcome has been determined. Pregnancy will be recorded as an AE in all cases. It will be qualified as an SAE only if it fulfils criteria.

## Obligations of Sponsor

During the course of the study, the Sponsor will report in an expedited manner all SAEs that are both unexpected and at least reasonably related to the study medication, to the investigators, to the Authorities, and to the Medicines and Healthcare products Regulatory Agency (MHRA) / Local Research Ethics Committee (LREC) as appropriate.

## List of criteria for discontinuation of study medication

The patient may be withdrawn from the study if any of the following events occur:

- Significant intolerance of study medication
- Investigator decides that it is in the best interests of the patient to terminate his/her participation in the study
- Poor patient compliance and/or major protocol deviation
- Patient wishes to terminate his/her participation in the study

The patients may withdraw from the study at any time irrespective of the reason. Given the short nature of the study, temporary discontinuations of study medication are not planned. The reason for medication discontinuation will be documented in the case record form.

## Handling of patients after treatment discontinuation

Patients withdrawn from the study will continue to be followed up according to the study protocol to the scheduled date of study completion, or to recovery or stabilisation of a followed-up AE, whichever comes last. Routine medical and surgical care of withdrawn patients will continue as planned. The investigator will make every effort to contact the patient in the case of a patient not attending a planned review appointment.

## Pregnancy

In the highly unlikely event that a subject becomes pregnant the study medication will be discontinued and the DMC and Sponsor informed. Pregnancy will be recorded as an adverse event in all cases. It will be qualified as an SAE only if it fulfils SAE criteria.

## List of criteria for premature discontinuation of the study

The study may be discontinued prematurely by the Sponsor, the DMC or the Principal Investigator if:

- As a result of the interim analysis or any other event during the study there is doubt as to the risk/benefit ratio
- The results of the trial do not appear to be scientifically convincing to the Sponsor
- The aim of the study has become outdated or is no longer of interest
- There is a breach by the investigator of a fundamental obligation under this agreement, including but not limited to breach of the Study Protocol, breach of the applicable laws and regulations or breach of the ICH guidelines for Good Clinical Practice

In all cases the LREC, MHRA and Health Authority will be informed.

# Study Procedures

## Visit schedule

Subjects will be identified and recruited as described above. The case record form will be completed, vital signs documented, an ECG performed and baseline blood samples taken at the time of the first visit, prior to randomisation. Patients will be reviewed in a private room in the surgical outpatient department.

A second review is planned after seven days of study medication. This semi-structured interview will be conducted in person and a blood test will be taken to check for haematological and biochemical parameters. Any subject who experiences any self-reported adverse events will be reviewed in person at the earliest opportunity.

A review is planned at the end of the two week period of study medication. In addition to a clinical review including recording of vital signs, repeat blood samples will be taken and an ECG performed. A further brief review for the purpose of determining any adverse events that may have occurred after the end of the period of treatment with the study drug will be performed during the operative inpatient stay (or at a relevant timepoint in the case of cancellation of surgery). **A final blood sample will be taken approximately 28 days after the date of surgery, at a routine postoperative clinic appointment.**

Participants will be provided with contact details for the investigators. Additional reviews will be arranged, if necessary, at the participants’ request.

It is anticipated that the study medication will be started fifteen days before the scheduled surgery to co-ordinate the end-of treatment study assessment with the pre-operative admission assessment.

## Treatment group assignation

Subjects recruited to the study will be randomised to receive either artesunate or placebo in equal proportions. Randomisation will be by computer-generated code, supplied in opaque envelopes by the study statistician. Copies of the key to the randomisation codes will be held by the Clinical Trials Pharmacist only. The code will only be broken in exceptional circumstances, when knowledge of the investigational drug is essential for medical treatment of the patient.

## Data collection

Data will be stored in anonymised form in a password-protected database in locked rooms. We will use Filemaker Pro™ as our relational database storage programme. Study documentation will be stored for ten years after the completion of the study.

Basic demographic and clinical information will be gathered at the time of the initial interview and by review of the medical notes if needed. The following information will be recorded for all subjects (see Appendix 2):

- Demographic details: Date of enrolment, age, gender, ethnicity, contact details for participant and GP
- Clinical data: symptomatology, risk factors for development of CRC, weight, height, vital signs
- Findings at endoscopy
- Imaging: findings of ultrasound, CT and/or MRI (performed as clinically indicated)
- Disease staging: TNM staging prior to surgery and definitive histological staging (TNM, Duke’s stage)
- Histology: routine histological grading, apoptosis count and specialist assays (see below)
- Non-study drug treatment: a full record of all non-study drugs taken will be kept
- Study-drug treatment: compliance with study medication, all adverse events, serious or otherwise

The following blood samples will be taken and analysed:

- Baseline: full blood count (FBC), urea and electrolytes, liver function tests (LFT), glucose, carcinoembryonic antigen (CEA)
- Mid treatment: FBC, urea and electrolytes, LFT
- End of treatment: FBC, urea and electrolytes, LFT
- Serum and plasma will be taken at baseline and end of treatment and stored at -80°C for proteomic analysis within one hour of phlebotomy
- Any other blood or clinical sample at any time if deemed necessary by the Investigators in case of a suspected adverse event
- **Peripheral blood mononuclear cells (PBMC) will be taken at baseline, at the end of treatment and after convalescence from surgery.**

Tissue samples will be taken at the time of surgery for analysis as described below.

# Outcome Measures

## Primary outcome measure - efficacy

The primary outcome measure for the treatment response study will be a significant difference in the proportion of cells that exhibit apoptosis between the 2 treatment groups (placebo and artesunate). As CRC is unlikely to exhibit significant apoptosis if untreated, >7% of cells with apoptotic features will be considered significant and consistent with drug effect. As there will be placebo-controlled subjects in this study (who will receive the conventional pre-operative standard of care), the proportion of subjects who have increased proportion of apoptotic cells in artesunate recipients will be compared with those receiving placebo.

The following sample size has been estimated by the statistician using Clinistat:

Estimated sample size for two-sample comparison of proportions

Test Ho: p1 = p2, where p1 is the proportion in population 1

and p2 is the proportion in population 2

Assumptions:

alpha = 0.0500 (two-sided)

power = 0.9000

p1 = 0.0500 (i.e. 1 patient or none will have apoptotic cells in placebo recipients).

p2 = 0.58 (i.e. 7 patients will have apoptotic cells in artesunate recipients).

n2/n1 = 1.00

Estimated required sample sizes:

n1 = 11

n2 = 11

This sample size has the power to detect a difference between apoptosis rates of 18% and 75%. This outcome measure will be scored without knowledge of intervention, as this is a double-blind investigation.

## Secondary outcome measures - tolerability

A secondary clinical outcome measure will be the tolerability of artesunate compared with placebo. Tolerability will be assessed according to conventional criteria used in clinical trials, and scored on standardised pro-formas.

Comparisons will also be made between baseline haematological and biochemical variables (FBC, LFT, urea and electrolytes), and these measures repeated once treatment stops and before surgery.

## Other histological assessments

These will be detailed assessments including those of tumour markers and other surface markers in routine use at St. George’s. Biopsies will be assessed specifically for specialised assays, carried out in Professor Thomas Efferth’s laboratory (Heidelberg) and will include:

- Determination of apoptosis using TUNEL assay (using a commercially available kits) – this is the primary endpoint of this study
- Immunohistochemical analyses of paraffin-embedded tumor sections with particular assessment of vascular endothelial growth factor (VEGF), *c-myc* status and EGF-receptor status
- Determination of microvessel density
- Proliferative activity (Ki-67 staining)

Material will be obtained at the time of resection of the tumour for routine histological purposes, and surplus material will be used for the histological studies listed. Several small pieces of tumour material (1 mm^3^) will also be collected into RNA later™ (Trizol), or without preservative and stored at

-70°C or below for expression studies using microarrays, and other genomic analyses.

An aliquot of serum will be stored on admission and just before surgery, for proteomic analyses (carried out at St. George’s). **PBMC typing, and the effect of artesunate on expression of cell surface markers will be assessed.**

## Interim analysis

An interim analysis comprising efficacy and tolerability criteria will be performed once the first ten patients have completed the study.

## Blinding

All assessments of primary or secondary endpoints will be undertaken by investigators blinded to treatment group.

## Statistics

All analyses will be made on an intention to treat basis. Statistical significance will be assumed at a 5% level.

# Ethical and regulatory standards

This study will be conducted in accordance with the principles laid down in the Declaration of Helsinki, and the International Conference on Harmonisation guidelines for Good Clinical Practice. Access to the data will be according to the Data Protection Act.

All patients will be fully informed by the investigators of all pertinent information to the trial in language and terms that they are able to understand. A written Patient Information Sheet (Appendix 3), approved by the Wandsworth LREC and the Sponsor will be provided as will an opportunity to ask questions of the investigators. Participation in the trial will not occur until a standard, approved Informed Consent Form has been signed and dated.

Approval for the study is being sought from the Wandsworth LREC of the National Research Ethics Service, the St George’s Healthcare NHS Trust Research and Development Office and the MHRA.

The relevant regulatory authorities including the DMC will have full access to all study documentation at all times.

# Miscellaneous Considerations

## Financing and Insurance

Artesunate and placebo are being provided free of charge by Dafra Pharma. The study is funded from Prof Krishna’s and Professor Kumar’s Research Funds.

## Insurance Compensation

Indemnity will be provided through the schemes operated by St George’s, University of London according to standard agreements.

## Publication Policy

Investigators agree not to make any publication, communication or release pertaining to the results of the study without the prior written permission of the Principal Investigator.

# Study Personnel

## Scientific Advisory Panel

Prof Devinder Kumar (Principal investigator)

Department of General Surgery, St George’s Healthcare NHS Trust, London

Tel: 020 8725 1301

e-mail: devinder.kumar@stgeorges.nhs.uk

Prof Sanjeev Krishna (Co-Principal investigator)

Centre for Infection, Division of Cellular and Molecular Medicine, St George’s, University of London

Tel: 020 8725 5836

Tel (put of hours): 07931 901724

e-mail: s.krishna@sgul.ac.uk

Prof Thomas Efferth,

German Cancer Research Center, Im Neuenheimer Feld 280

69120 Heidelberg

Germany

Tel: +49 6221 423426

e-mail: t.efferth@dkfz-heidelberg.de

Dr Herwig Jansen,

Dafra Pharma, Slachthuisstraat 30/7

B-2300 Turnhout, Belgium

Tel: + 321 461-7820

e-mail: herwig.jansen@dafra.be

## Research Fellows

Nicholas Katsoulas

30B Thornton Avenue

Chiswick

London

W4 1QG

Tel: 07958 380 322

e-mail: [nkatsoulas@gmail.com](mailto:nkatsoulas@gmail.com)

Senthil Kumar Ganapathy

Honorary Research Fellow

CDS - Non-Staff

Tel: 0787 067 7881

sganapat@sgul.ac.uk

## Clinical Trials Pharmacists

Ms Debbie Rolfe,

Pharmacy, St George’s Healthcare NHS Trust, London

Tel: 020 8725 1294

e-mail: [Debbie.rolfe@stgeorges.nhs.uk](mailto:Debbie.rolfe@stgeorges.nhs.uk)

Ms Joanne Harding,

Medicines Information, St George’s Healthcare NHS Trust, London

Tel: 020 8725 3685

e-mail: [Joanne.harding@stgeorges.nhs.uk](mailto:Joanne.harding@stgeorges.nhs.uk)

## Data Monitoring Committee

Prof Peter Kremsner (chair)

Institute for Tropical Medicine, University Hospital Tübingen

Tel: (49) 7071 29 8 71 79

e-mail: peter.kremsner@uni-tuebingen.de

Dr Jan Poloniecki (statistician)

Department of Community Health Sciences, St George’s, University of London

Tel: 020 8725 2795

e-mail: j.poloniecki@sgul.ac.uk

Mr Paul Toomey

Department of Surgery, Epsom and St Helier NHS Trust, Surrey

Tel: 01372 735470

e-mail: paultoomey@btinterent.com

## Sponsor’s representative

Mr Javier Moreno Farre,

Clinical Trials Monitor

Joint Research Office

St George's, University of London

Ground Floor, Hunter Wing

Cranmer Terrace

London

SW17 0RE

Tel: 02087253784

Fax: 020 8725 3426

e-mail: jmoreno@sgul.ac.uk

# References

[1] Meyerhardt J, Saunders M, eds. Colorectal Cancer. 1st. ed. China: Elsevier Mosby 2007.

[2] Golfinopoulos V, Salanti G, Pavlidis N, Ionnidis P. Survival and disease-progression benefits with treatment regimens for advanced colorectal cancer: a meta-analysis. The Lancet Oncology. 2007;in press.

[3] Hien TT. An overview of the clinical use of artemisinin and its derivatives in the treatment of falciparum malaria in Viet Nam. Trans R Soc Trop Med Hyg. 1994.

[4] Ashley EA, White NJ. Artemisinin-based combinations. Curr Opin Infect Dis. 2005 Dec;18(6):531-6.

[5] Kremsner PG, Krishna S. Antimalarial combinations. The Lancet. 2004;364:285-94.

[6] Efferth T, Rucker G, Falkenberg M, Manns D, Olbrich A, Fabry U, et al. Detection of apoptosis in KG-1a leukemic cells treated with investigational drugs. Arzneimittelforschung. 1996 Feb;46(2):196-200.

[7] Efferth T, Dunstan H, Sauerbrey A, Miyachi H, Chitambar CR. The anti-malarial artesunate is also active against cancer. Int J Oncol. 2001 Apr;18(4):767-73.

[8] Efferth T. Willmar Schwabe Award 2006: antiplasmodial and antitumor activity of artemisinin--from bench to bedside. Planta Med. 2007 Apr;73(4):299-309.

[9] Li LN, Zhang HD, Yuan SJ, Tian ZY, Wang L, Sun ZX. Artesunate attenuates the growth of human colorectal carcinoma and inhibits hyperactive Wnt/beta-catenin pathway. Int J Cancer. 2007 Sep 15;121(6):1360-5.

[10] Berger TG, Dieckmann D, Efferth T, Schultz ES, Funk JO, Baur A, et al. Artesunate in the treatment of metastatic uveal melanoma--first experiences. Oncol Rep. 2005 Dec;14(6):1599-603.

[11] Singh N, Verma K. Case report of a laryngeal squamous cell carcinoma treated with artesunate. Archives of Oncology. 2002;2002:279-80.

[12] Singh NP, Panwar VK. Case report of a pituitary macroadenoma treated with artemether. Integr Cancer Ther. 2006 Dec;5(4):391-4.

[13] Golenser J, Waknine JH, Krugliak M, Hunt NH, Grau GE. Current perspectives on the mechanism of action of artemisinins. Int J Parasitol. 2006 Dec;36(14):1427-41.

[14] Krishna S, Woodrow CJ, Staines HM, Haynes RK, Mercereau-Puijalon O. Re-evaluation of how artemisinins work in light of emerging evidence of in vitro resistance. Trends Mol Med. 2006 May;12(5):200-5.

[15] Mu D, Chen W, Yu B, Zhang C, Zhang Y, Qi H. Calcium and survivin are involved in the induction of apoptosis by dihydroartemisinin in human lung cancer SPC-A-1 cells. Methods Find Exp Clin Pharmacol. 2007 Jan-Feb;29(1):33-8.

[16] Zhou HJ, Wang WQ, Wu GD, Lee J, Li A. Artesunate inhibits angiogenesis and downregulates vascular endothelial growth factor expression in chronic myeloid leukemia K562 cells. Vascul Pharmacol. 2007 Aug-Sep;47(2-3):131-8.

[17] Eckstein-Ludwig U, Webb R, I.D.A. vG, J.M. E, A.G. L, M K, et al. Artemisinins target the SERCA of *Plasmodium falciparum*. Nature. 2003;424:957-61.

[18] Uhlemann AC, Cameron A, Eckstein-Ludwig U, Fischbarg J, Iserovich P, Zuniga FA, et al. A single amino acid residue can determine the sensitivity of SERCAs to artemisinins. Nat Struct Mol Biol. 2005 Jul;12(7):628-9.

[19] Krishna S, Planche T, Agbenyega T, Woodrow C, Agranoff D, Bedu-Addo G, et al. Bioavailability and preliminary clinical efficacy of intrarectal artesunate in Ghanaian children with moderate malaria. Antimicrob Agents Chemother. 2001;45(2):509-16.

[20] Adjuik M, Agnamey P, Babiker A, Borrmann S, Brasseur P, Cisse M, et al. Amodiaquine-artesunate versus amodiaquine for uncomplicated Plasmodium falciparum malaria in African children: a randomised, multicentre trial. Lancet. 2002 Apr 20;359(9315):1365-72.

[21] Simpson JA, Agbenyega T, Barnes KI, Perri GD, Folb P, Gomes M, et al. Population Pharmacokinetics of Artesunate and Dihydroartemisinin following Intra-Rectal Dosing of Artesunate in Malaria Patients. PLoS Med. 2006 Nov;3(11):e444.

[22] Scott RH, Mansour S, Pritchard-Jones K, Kumar D, MacSweeney F, Rahman N. Medulloblastoma, acute myelocytic leukemia and colonic carcinomas in a child with biallelic MSH6 mutations. Nat Clin Pract Oncol. 2007 Feb;4(2):130-4.

[23] Evans C, Dalgleish AG, Kumar D. Review article: immune suppression and colorectal cancer. Aliment Pharmacol Ther. 2006 Oct 15;24(8):1163-77.

[24] Evans C, Morrison I, Heriot AG, Bartlett JB, Finlayson C, Dalgleish AG, et al. The correlation between colorectal cancer rates of proliferation and apoptosis and systemic cytokine levels; plus their influence upon survival. Br J Cancer. 2006 May 22;94(10):1412-9.

# Appendix 1: Study design

Randomisation

Artesunate 200mg od for 14 days

Placebo for 14 days

**Referral, diagnosis, surgery planned**

Day 0

Day7

Day14

Recruitment interview and randomisation

<62 days

Midpoint assessment

End of treatment assessment

**Surgery**

(post-treatment review)

# Appendix 2: Sample Case Record Form

#

#
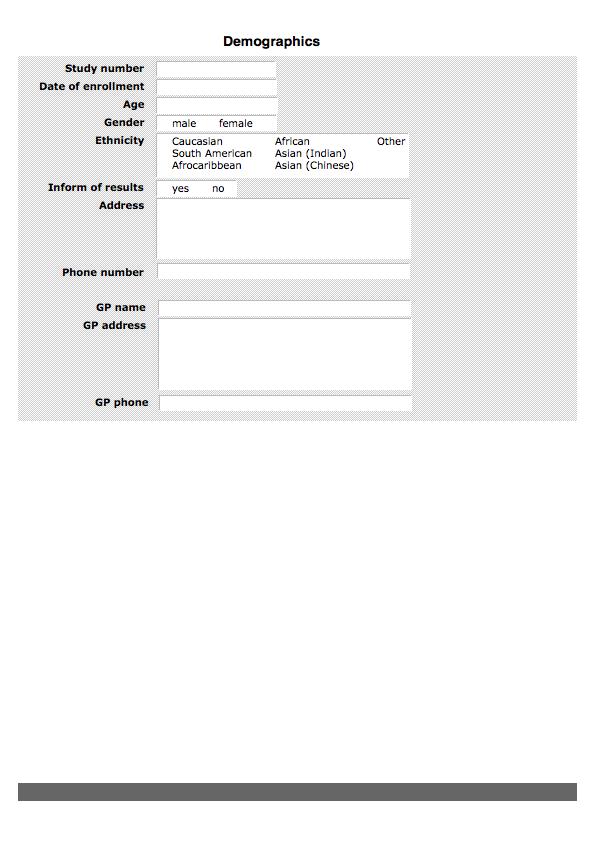


#
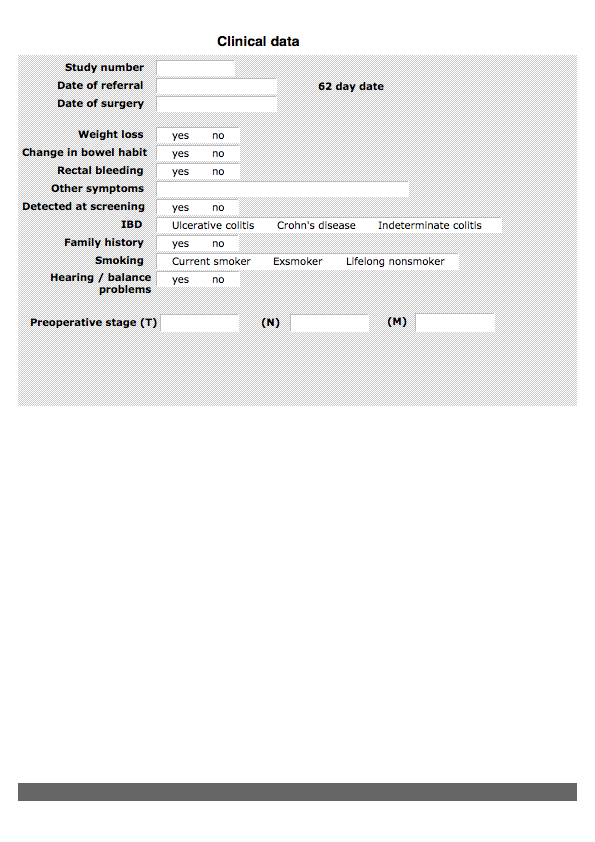


#
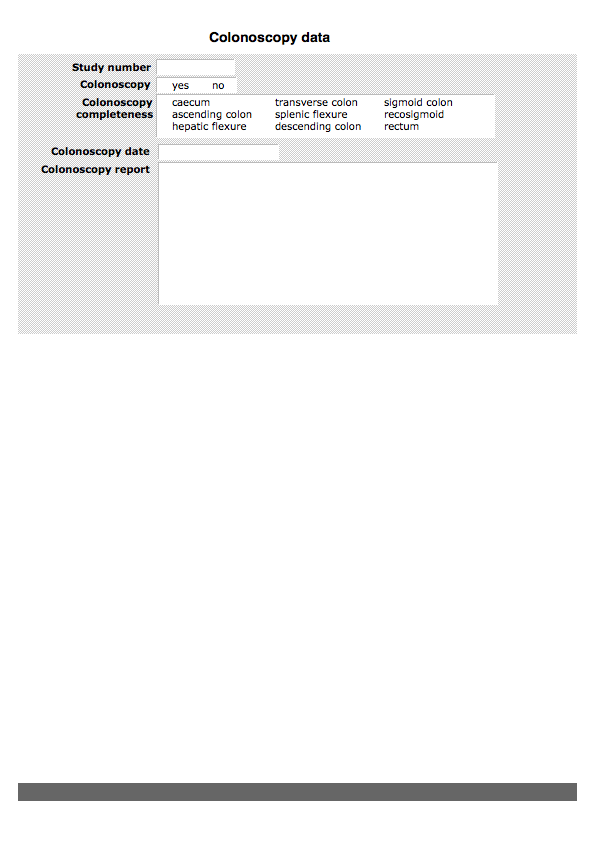


#
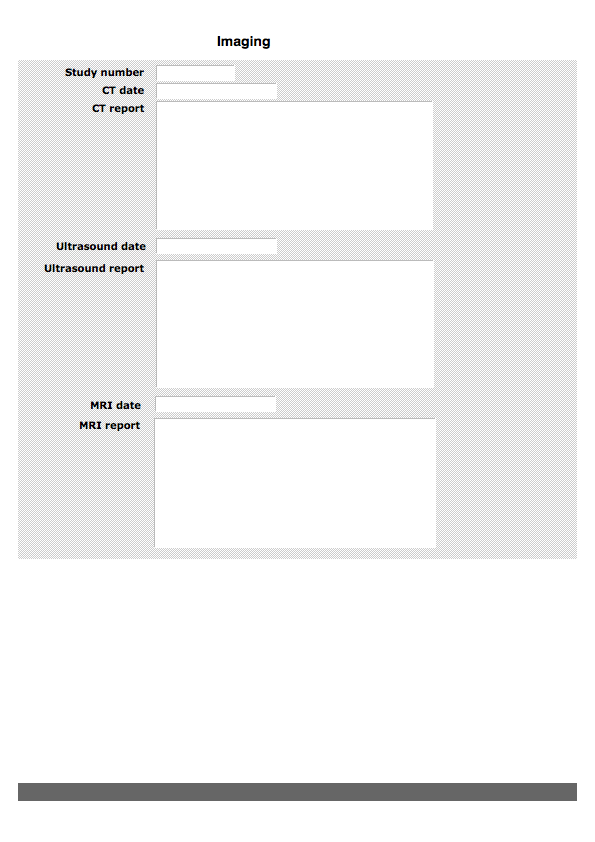


#
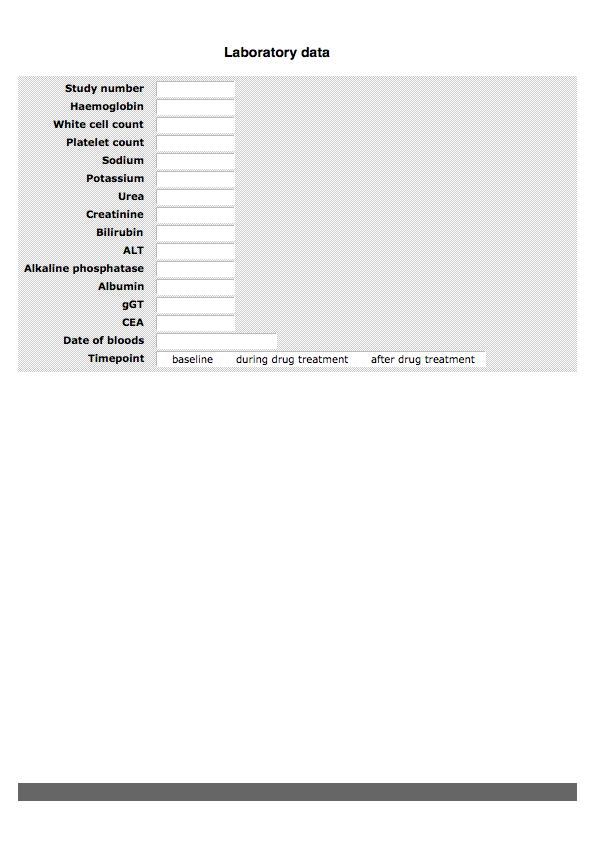


#
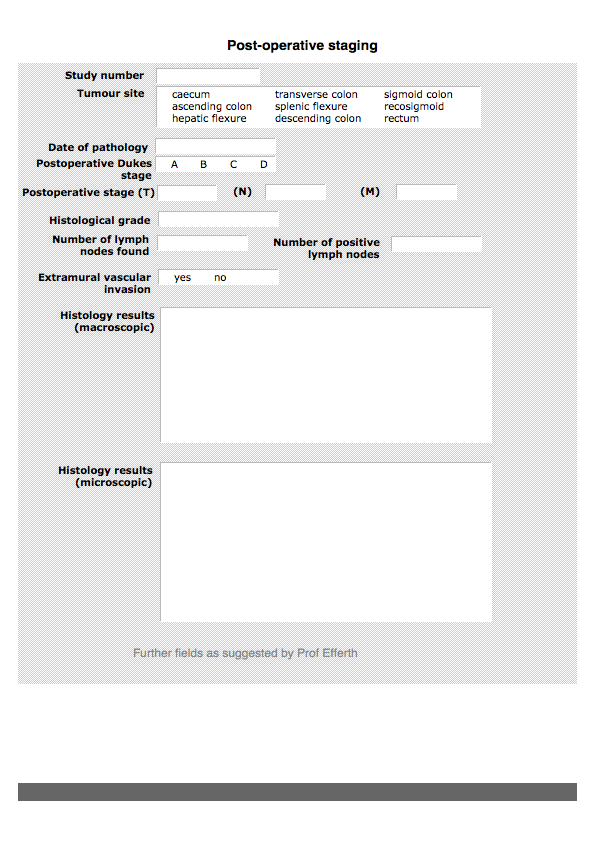


#
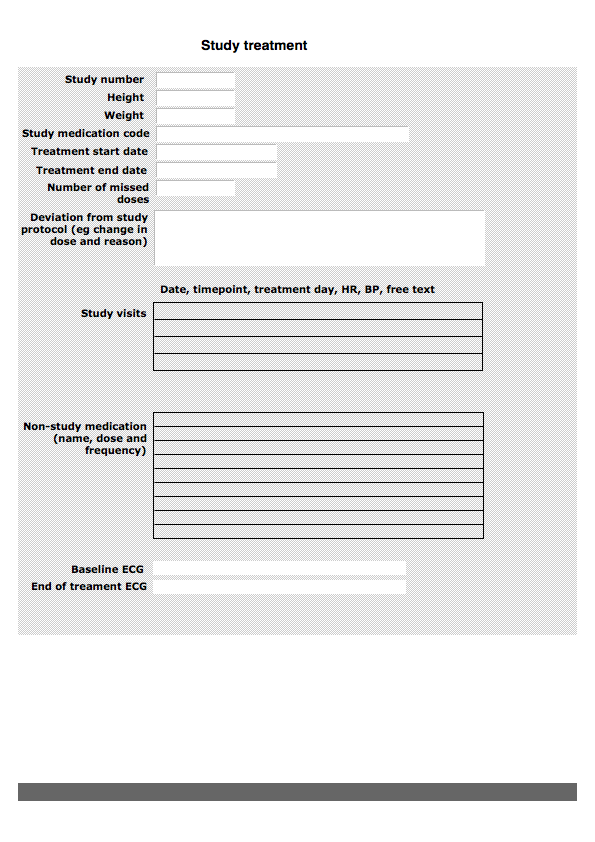


#
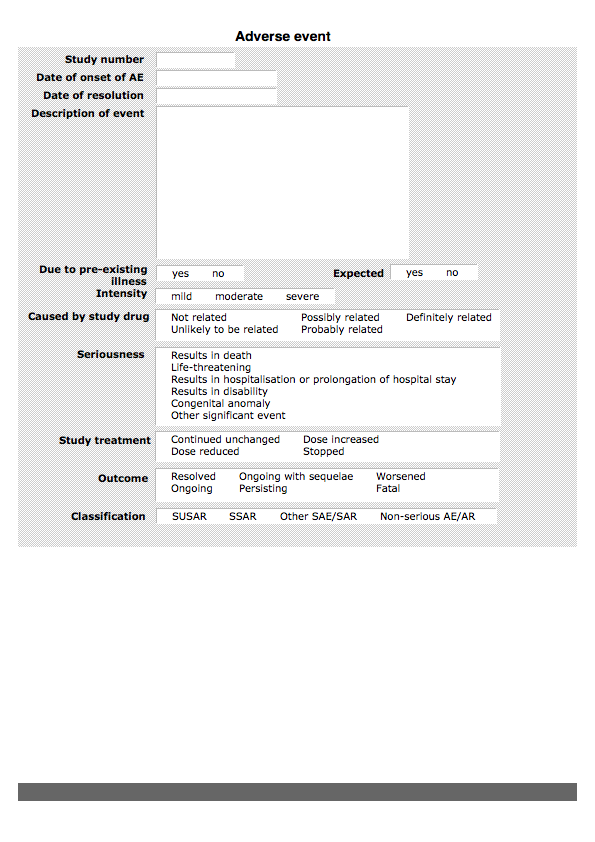


#
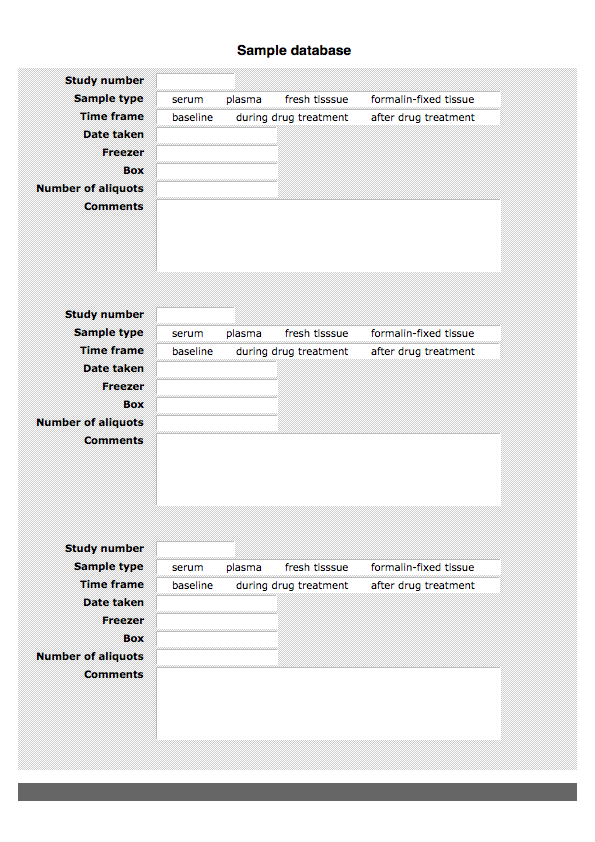


# Appendix 3: Patient Information Sheet

**Patient Information Sheet** *(May 2010 version 4)*

**Phase I study of oral artesunate in colorectal cancer**

**Part 1**

You are being invited to take part in a research study. Before you decide it is important for you to understand why the research is being done and what it will involve. Please take time to read the following information carefully and discuss it with others if you wish.

- Part 1 tells you the purpose of this study and what will happen to you if you take part.
- Part 2 gives you more detailed information about the conduct of the study.

Ask us if there is anything that is not clear or if you would like more information. Take time to decide whether or not you wish to take part. Thank-you for reading this.

***What is the purpose of the study?***

There is evidence that artesunate, which is a drug that millions of adults and children have taken for the treatment of malaria, causes the death of cancer cells (apoptosis) when given to animals. Artesunate has not previously been given to humans for this reason. We would like to know whether a short course of the drug can induce colon cancer cells to die. We also need to ensure that patients with cancer can tolerate the drug. Although recommended by the World Health Organisation for the treament of malaria, artesunate is not currently licensed for use in the UK and is only being used in research.

***Why have I been chosen?***

You are waiting to have curative surgery for bowel cancer. We will be recruiting about 20 patients in your position.

***Do I have to take part?***

It is up to you whether or not to take part. We will discuss the study and give you a copy of this information sheet. If you decide to take part we will ask you to sign a consent form. You are still free to withdraw at any time and without giving a reason. This will not affect the care you receive.

***What will happen if I decide to take part?***

Because patients with cancer have not received artesunate before, we will randomly allocate you to receive either artesunate or a placebo for two weeks. A placebo is a dummy tablet that looks identical to the genuine medicine but contains no active ingredient. We will ask you some baseline questions about your medical history and will examine your clinical notes for details about the tests that you have had. We will then need to take a blood sample – this will usually be done at the time you have routine blood tests at the clinic. We will review you to ensure that the medication is not causing any side-effects and will repeat the blood tests during and at the end of the artesunate course**, and after you have had your surgery**. The blood tests are performed to monitor the artesunate (see below), to detect proteins in the blood that might predict how the cancer responds to treatment**, and to measure the levels of the natural cancer-fighting white blood cells in the blood**. After you have had your surgery we will examine the tumour. After we have performed routine examination of the tumour, a portion will be sent to collaborators in Germany who will count the number of cancer cells that show evidence of cell death. We will also examine the DNA in the cancer cells to understand the way that the cancer has mutated.

***What will I have to do?***

You will have to take four tablets daily for fourteen days. You should continue to take any other medication unchanged. We will need to review you after you have taken the study drug for a week to ensure that you are not having any side-effects. If you are well, we will do this over the phone. We will need to review you in person once you have completed the course of treatment. We will also give you a phone number so that you can contact us if you are concerned about any aspect of the study.

***What is the drug that is being tested?***

Artesunate is a drug that has been taken by millions of people worldwide for the treatment of malaria. The dose that we will be using is 200mg orally once daily for 14 days. This is approximately the same as the dose given to patients with malaria, although the normal treatment course in this case is 7 to 10 days. Artesunate interacts with a few other medications but we will not recruit patients who are taking these drugs. Please tell us if you are taking drugs to prevent palpitations, antidepressants, antipsychotic drugs, antibiotics or treatment for fungal infections.

***What are the possible disadvantages and risks of taking part including drug side-effects?***

You are due to receive the best treatment for colon cancer (surgery) and participation in this study will not affect this or delay its timing. About 5% of people who take artesunate experience stomach upsets (including nausea, diarrhoea or abdominal cramps). Rashes occur rarely, as do skin tingling, palpitations and allergic reactions. Slight changes in blood count and liver blood tests may occur but these are reversible on stopping the drug. Hearing problems have developed in a very small number of patients who have received high doses of artesunate. We will be monitoring your health closely to ensure that any severe side-effects do not occur.

***Harm to the unborn child***

Artesunate may harm the unborn child if taken during the first three months of pregnancy. For this reason, we will not recruit any patients who are known to be pregnant. Although we consider it highly unlikely that you will become pregnant during the period that you are taking the study medication, you must agree to use a reliable form of contraception during this time (eg oral contraceptive and condom, intra-uterine device (IUD, “coil”) and condom, diaphragm with spermicide and condom). If you do discover that you are pregnant during the period of the study, we would ask you to tell the study doctor immediately (in addition to your surgeon) so we can help decide appropriate action.

***What are the possible benefits of taking part?***

We cannot promise that this study will help you but hope that the information we gain will help improve the care of people with colon cancer.

***What happens when the study stops?***

After surgery you will continue with normal clinical care pathways. There are no plans to continue artesunate treatment after surgery.

***What if there is a problem?***

Any complaint about the way you have been dealt with during the study or any possible harm you might suffer will be addressed. The detailed information on this is given in Part 2.

***Will my taking part in this study be kept confidential?***

All information that is collected about you during the course of the research will be kept strictly confidential. The details are included in Part 2.

***This completes Part 1 of the Information Sheet. If the information in Part 1 has interested you and you are considering participation, please continue to read the additional information in Part 2 before making any decision.***

**Part 2**

***What if relevant new information becomes available?***

Sometimes we get new information about the treatment being studied. If this happens, your research doctor will tell you and discuss whether you should continue in the study. If you decide not to carry on, your research doctor will make arrangements for your care to continue. If you decide to continue in the study he may ask you to sign an updated consent form. If this happens, your research doctor might consider you should withdraw from the study. He/she will explain the reasons and arrange for your care to continue. If the study is stopped for any other reason, we will tell you and arrange your continuing care.

***What will happen if I don’t want to carry on with the study?***

If you withdraw from the study, we will destroy all your identifiable samples, but we will need to use the data collected up to your withdrawal.

***What if there is a problem?***

If you have a concern about any aspect of this study, contact the study doctors using the details given below. We will do our best to answer your questions. In the unlikely event that you are harmed as a consequence of the research and this is due to someone’s negligence then you may have grounds for a legal action for compensation against St George’s, University of London but you may have to pay your legal costs. The NHS Indemnity scheme does not provide cover for no-fault compensation. The normal NHS complaints mechanisms will still be available to you. Concerns regarding your normal medical care should be addressed to the surgical team.

***Will my taking part in this study be kept confidential? What will happen to my any samples I give?***

Any information about you or samples taken will have your name removed so that you cannot be recognised. Any data or samples will be stored in password protected files in locked rooms that only the researchers (and relevant regulatory bodies) have access to. We will inform your hospital consultant and GP if you decide to take part in the study.

***What will happen to the results of the research?***

Eventually we plan to publish the results in a scientific journal. You will not be identified in any report/publication. We will send you a copy of the findings, if you would like.

***Who is organising and funding the research?***

Dafra Pharma has provided the study drug free of charge but is not involved in the organisation or running of the study. The study is funded from Prof Krishna’s Research Fund. None of the doctors or researchers involved have received additional payment.

***Who has reviewed the study?***

The Wandsworth Local Research Ethics Committee has reviewed this study.

Thank you for considering taking part!

***Contacts for further information.***

Nicholas Katsoulas

30B Thornton Avenue

Chiswick

London

W4 1QG

Tel: 07958 380 322

e-mail: [nkatsoulas@gmail.com](mailto:nkatsoulas@gmail.com)

Senthil Kumar Ganapathy

Honorary Research Fellow

CDS - Non-Staff

Tel: 0787 067 7881

sganapat@sgul.ac.uk
